# Kindlin-2 regulates the oncogenic activities of integrins and TGF-β in triple-negative breast cancer progression and metastasis

**DOI:** 10.1038/s41388-024-03166-2

**Published:** 2024-09-19

**Authors:** Neelum Aziz Yousafzai, Lamyae El Khalki, Wei Wang, Justin Szpendyk, Khalid Sossey-Alaoui

**Affiliations:** 1 MetroHealth System, Cleveland, OH USA; 2 Case Western Reserve University, Cleveland, OH USA; 3 Case Comprehensive Cancer Center, Cleveland, OH 44109 USA

**Keywords:** Breast cancer, Extracellular signalling molecules, Mechanisms of disease, Integrins

## Abstract

Kindlin-2, an adapter protein, is dysregulated in various human cancers, including triple-negative breast cancer (TNBC), where it drives tumor progression and metastasis by influencing several cancer hallmarks. One well-established role of Kindlin-2 involves the regulation of integrin signaling, achieved by directly binding to the cytoplasmic tail of the integrin β subunit. In this study, we present novel insights into Kindlin-2’s involvement in stabilizing the β1-Integrin:TGF-β type 1 receptor (TβRI) complexes, acting as a physical bridge that links β1-Integrin to TβRI. Loss of Kindlin-2 results in the degradation of this protein complex, leading to the inhibition of downstream oncogenic pathways. We used a diverse range of in vitro assays, including CRISPR/Cas9 gene editing, cell migration, 3D-tumorsphere formation and invasion, solid binding, co-immunoprecipitation, cell adhesion and spreading assays, as well as western blot and flow cytometry analyses, utilizing MDA-MB-231 and 4T1 TNBC cell lines. Additionally, preclinical in vivo mouse models of TNBC tumor progression and metastasis were employed to substantiate our findings. Our studies established the direct interaction between Kindlin-2 and β1-Integrin and between Kindlin-2 and TβRI. Disruption of these interactions, via CRISPR/Cas9-mediated knockout of Kindlin-2, led to the degradation of β1-Integrin and TβRI, resulting in the inhibition of oncogenic pathways downstream of both proteins, subsequently hindering tumor growth and metastasis. Treatment of Kindlin-2-deficient cells with the proteasome inhibitor MG-132 restored the expression of both β1-Integrin and TβRI. Furthermore, the rescue of Kindlin-2 expression reinstated their oncogenic activities in vitro and in vivo, while Kindlin-2 lacking domains involved in the interaction of Kindlin-2 with β1-Integrin or TβRI did not. This study identifies a novel function of Kindlin-2 in stabilizing the β1-Integrin:TβRI complexes and regulating their downstream oncogenic signaling. The translational implications of these findings are substantial, potentially unveiling new therapeutically targeted pathways crucial for the treatment of TNBC tumors.

## Introduction

Breast Cancer (BC) ranks as the second leading cause of cancer-related deaths among women in the United States, with more than 300,000 new cases reported annually and 42,250 lives lost [1]. The acquisition of the metastatic phenotypes accounts for approximately 90% of BC-related deaths [2]. Metastatic BC, typically incurable, imposes a median survival of only 1.5 to 3 years for affected patients. Clinically, about 30% of BC patients initially diagnosed with early-stage, noninvasive disease progress to late-stage, metastatic disease, significantly limiting treatment options and resulting in dismal clinical outcomes [3]. This challenge is exacerbated by the heterogeneous nature of BCs, comprising genetically distinct subtypes [4–6], with triple-negative BCs (TNBCs) standing out as particularly lethal due to their highly metastatic behavior and rapid recurrence [7–11]. TNBCs lack expression of hormone receptors (ER-α and PR) and ErbB2/HER2 [7–11], which imposes a hurdle for FDA-approved targeted drug therapies. Additionally, TNBCs often develop resistance to standard-of-care treatments through unidentified mechanisms. Hence, preventing TNBC progression and recurrence emerges as a critical strategy to significantly enhance the clinical course for TNBC patients.

Kindlins, a small gene family of FERM domain-containing adapter proteins, comprising three members, with Kindlin-2 being the most widely expressed [12, 13]. Dysregulated Kindlin expression is linked to various human pathologies, including cancer [13, 14]. In particular, Kindlin-2 plays a pivotal role in BC progression, promoting metastasis and invasion [15–17]. Kindlin-2 regulates the growth and progression of BC tumors by activating CSF-1-mediated macrophage infiltration, thereby promoting metastatic progression [15]. Its involvement extends to regulating tumor growth and progression by enhancing Wnt signaling through complex formation with β‐catenin and TCF4, and contributing to Src-mediated tyrosine phosphorylation of androgen receptor [18, 19]. Therefore, Kindlin-2 has been established as a major regulator of several hallmarks of cancer [20]. Published studies from our group [12, 21–23] further established Kindlin-2 as a major driver of the invasion-metastasis cascade in TNBC, influencing epithelial-to-mesenchymal transition (EMT), cancer cell senescence, chemotherapeutic sensitization, and actin-mediated integrin outside-in signaling [12, 21–23]. Noteworthy contributions of Kindlin-2 to BC pathogenicity include the regulation of HIF-1α-mediated activation of tumor angiogenesis, mitotic spindle assembly through inhibiting histone deacetylase 6, maintenance of α-tubulin acetylation, and stabilization of DNA methyltransferase 1 (DNMT1) [24–26]. Both in cancer cells and mammary glands, Kindlin-2 is established as a requirement for BC tumor development and progression in transgenic mice [27]. We also established Kindlin-2’s pivotal role in regulating TNBC progression and metastasis through CSF-1/EGF paracrine signaling and upstream TGF-β [15]. Of particular interest is the interaction between Kindlin-2 and TGF-β Type One Receptor (TβRI), elucidated by Wei and colleagues [28].

Given Kindlin-2’s role as a coactivator of integrin activities and its interaction with TβRI, our study investigated the impact of inhibiting these interactions on downstream signaling of both Integrins and TβRI, and their role in TNBC tumor progression and metastasis. Our findings confirm direct interactions between Kindlin-2, β1-Integrin, and TβRI, with Kindlin-2 crucial for stabilizing the β1-Integrin:TβRI protein complexes. Loss of Kindlin-2 expression leads to degradation of both β1-Integrin and TβRI proteins, which can be rescued by re-expression of Kindlin-2. Importantly, loss of Kindlin-2 expression inhibits downstream signaling pathways of both β1-Integrin and TβRI. The biological significance of Kindlin-2-mediated stabilization of the β1-Integrin:Kindlin-2:TβRI protein complexes is reflected in the inhibition of the oncogenic behavior of TNBC tumors lacking Kindlin-2, β1-Integrin, or TβRI, both in vitro and in in vivo mouse models.

In summary, our findings unveil a novel role for Kindlin-2 in simultaneously regulating the oncogenic activities of both β1-Integrin and TβRI by stabilizing the β1-Integrin: Kindlin-2:TβRI complex; an insight that holds promise for advancing our understanding and potential therapeutic interventions in TNBCs.

## Results

### Kindlin-2 is overactivated in triple-negative breast cancer tumors

Prior studies have established the role of Kindlin-2 as a major driver of tumor progression and metastasis in several cancers including the one that originates in the breast [14]. Published studies form our group and others have also shown that Kindlin-2 is involved in the activation of the oncogenic behavior of TNBC tumors, both in vitro and in vivo [15, 16, 19, 21–24, 26, 27, 29]. Interrogation of a BC tumor microarray generated from BC specimens representing the different BC subtypes [15, 30], showed high levels of Kindlin-2 staining in advanced BC stages (Fig. S1A, B). Kindlin-2 staining score was significantly (*p* < 0.05) higher in BC tumors compared to normal breast tissues (Fig. S1B). More importantly, Kindlin-2 staining score was significantly (*p* < 0.05) higher in hormone receptor-negative and Her2-negative tumors, e.g. TNBC subtype (Fig. S1B), a tread that was also found in human and murine cell lines of TNBC nature [15, 30]. These findings were further confirmed by interrogation of public BC datasets from Oncomine (www.oncomine.org), where we found Kindlin-2 mRNA expression levels to be significantly (*p* < 0.001) higher in BC tumors compared to normal breast tissues [15]. Furthermore, interrogation of the KM-Plotter BC database (https://kmplot.com/analysis/) showed increased mRNA expression levels of Kindlin-2 correlate with poor disease outcome in patients with BC tumors, irrespective of subtype (Fig. S1C and [15]), and specifically in patients with TNBC tumors, at the mRNA levels (Fig. S1D), and at the protein levels (Fig. S1E). These findings, together with our published studies [15, 16, 21–23, 29], support the key role that Kindlin-2 plays in the pathology of BC tumors, in general, and in TNBC tumors, in particular.

One of the major functions of Kindlin-2 is the activation of the inside-out signaling of integrins through its physical interaction with the cytoplasmic tail of several integrin β-subunits, including β1-Integrin (Reviewed in [25, 31]). Additionally, we previously showed that Kindlin-2 activates the CSF1/EGF paracrine oncogenic loop in TNBC through the regulation of TGF-β signaling [15]. Interestingly, a study by Wei et al. [28] showed that Kindlin-2 also interacts with the cytoplasmic region of TGF-β type one receptor (TβRI). Accordingly, our published studies have shown that Kindlin-2 plays a major role in the regulation of TNBC tumor progression and metastasis through the regulation of the oncogenic activities of both Integrins and TGF-β. Based on this information we sought to investigate the molecular mechanisms that regulate the Kindlin-2 interaction with both β1-Integrin and TβRI, and the role of these interactions in the regulation TNBC tumor progression and metastasis.

### Kindlin-2 interacts with both β1-Integrin and TβRI

**Fig. 1:**
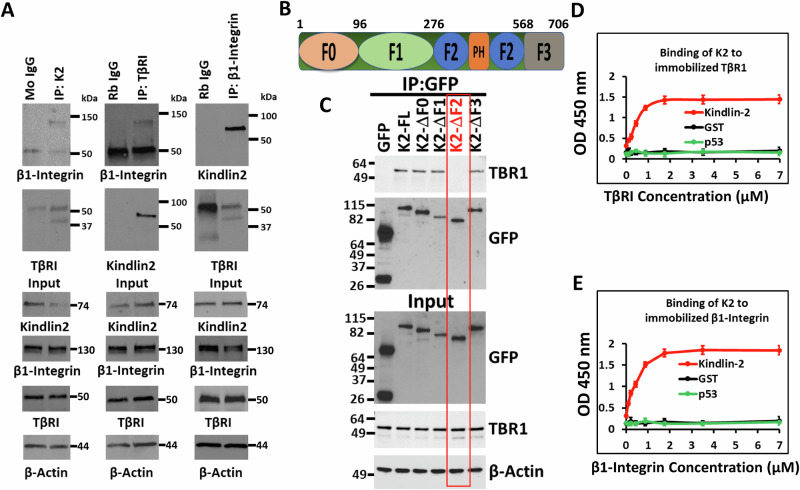
Kindlin-2 interacts with both β1-Integrin and TβRI. **A** Co-immunoprecipitation of total protein lysates from MDA-MB-MB-231 cells showing interactions between endogenous K2, β1-Integrin and TβRI proteins. **B** Diagram representation of the different K2 domains. **C** Pull-down assays showing that the F2 domain of K2 is necessary for the interaction between K2 and TβRI. Solid binding assay monitoring the binding of recombinant Kindlin-2 to immobilized TβRI (**D**) or β1-Integrin (**E**).

Co-immunoprecipitation using total protein lysates from MDA-MB-231 TNBC cells showed Kindlin-2 immunocomplexes captured both TβRI and β1-Integrin (Fig. 1A, left panels), while TβRI immunocomplexes also readily captured both Kindlin-2 and β1-Integrin (Fig. 1A, middle panels), and β1-Integrin immunocomplexes readily captured Kindlin-2 and TβRI (Fig. 1A, right panels), thereby implicating Kindlin-2 as a potential adapter that coordinates the formation of TβRI and β1-Integrin complexes. In addition, pulldown assays identified the F2 domain within Kindlin-2 (Fig. 1B) as being necessary for the interaction between Kindlin-2 and TβRI (Fig. 1C). The interaction between Kindlin-2 and the cytoplasmic tail of β1-Integrin has already been established to be mediated via the Q^614^W^615^ amino acid doublet that resides within the F3 domain of Kindlin-2 [32]. Finally, solid binding assay monitoring the binding of recombinant Kindlin-2 to immobilized TβRI (Fig. 1D) or β1-Integrin (Fig. 1E), established a direct binding of Kindlin-2 to TβRI and β1-Integrin, thereby establishing a physical bridge between TβRI and β1-Integrin.

### Kindlin-2 is required for the stabilization of the β1-Integrin:Kindlin-2:TβRI protein complex

**Fig. 2:**
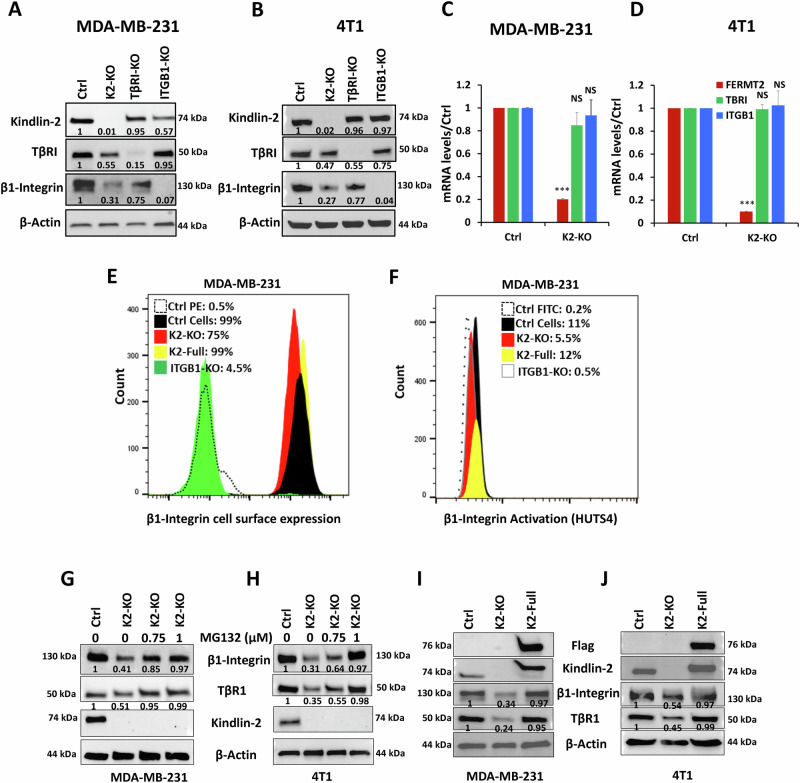
Kindlin-2 is required for the stabilization of the β1-Integrin:Kindlin-1:TβRI protein complex. MDA-MB-231 cells (**A**) and 4T1 cells (**B**) were subjected to CRISPR/Cas 9 mediated gene editing to knockout FERMT 2 (K2), ITGB1 (β1-Integrin) or TβRI genes, and their protein expression was assessed by Western Blot analysis. β-Actin is a loading control. qt-RT-PCR of mRNA expression levels of TβRI and β1-Integrin in the K2-KO MDA-MB-231 (**C**) and 4T1 (**D**) TNBC cells. **E**, **F** Flow cytometry histograms of the cell surface expression levels of β1-Integetrin (**E**) and its activated form (**F**) in K2-KO, *ITGB1*-KO and K2-full rescued K2-KO MDA-MB-231 cells. Western Blot analysis with indicated antibodies showing expressions of TβRI and β1-Integrin proteins in MDA-MB-231 K2-KO after treatment with MG132 (**G**, **H**) or after rescue of K2-full length (**I**, **J**). The numbers under each WB band represent the fold change in signal intensity with respect to its respective control band in each panel after normalization to the loading control signal. Data shown are representative of 3 replicates. Data are the mean ± SD (*n* = 3, *p < 0.05, Student’s *t*-test).

Probing further into the importance of Kindlin-2 in maintaining the integrity of β1-Integrin/Kindlin-2/TβRI protein complexes, we found loss of expression of Kindlin-2 (K2-KO) in MDA-MB-231 (Fig. 2A) or 4T1 (Fig. 2B) TNBC cells leads to the degradation of both TβRI and β1-Integrin proteins. mRNA expression levels of either TβRI or β1-Integrin were not significantly affected in the K2-KO MDA-MB-231 (Fig. 2C) or 4T1 (Fig. 2D) TNBC cells, suggesting that loss of expression of TβRI and β1-Integrin in the K2-KO cells was a result of protein degradation, but not at the mRNA transcription levels. We also used flow cytometry to measure the cell surface expression levels of β1-Integetrin and found loss of expression (KO) of Kindlin-2 resulted in inhibition of β1-Integrin expression at the surface of the cell membrane where it exerts its signaling functions (Fig. 2E). Over-expression of full-length Kindlin-2 (K2-full) in the K2-KO MDA-MB-231 cells restored cell surface expression of β1-Integrin to levels comparable to those found in the control cells (Fig. 2E, yellow histogram). We also used the HUTS4 assay that measures the active state of β1-Integrin [33], and found K2-KO, resulted in inhibition of activation levels of β1-Integrin (Fig. 2F), which could also be restored by over-expressing of full-length Kindlin-2 (K2-full) in the K2-KO cells (Fig. 2F, yellow histogram). In both experiments, we used β1-Integrin-KO as a control for both surface expression and activity. Moreover, treating the K2-KO MDA-MB-231 (Fig. 2G) or 4T1 cells (Fig. 2H) with the proteasome inhibitor MG132 resulted in the restoration of both TβRI and β1-Integrin proteins to levels found in the control cells, meanwhile overexpression of full-length Kindlin-2 in the K2-KO cells restored protein expression of both TβRI or β1-Integrin (Fig. 2I, J). Therefore, these data support the role of Kindlin-2 in maintaining the physical integrity of the TβRI:Kindlin-2:β1-Integrin protein complex.

### Loss of expression of either Kindlin-2, TβRI or β1-Integrin inhibits the in vitro oncogenic behavior of TNBC tumors, and re-expression of Kindlin-2 is sufficient for the restoration of these oncogenic activities

**Fig. 3:**
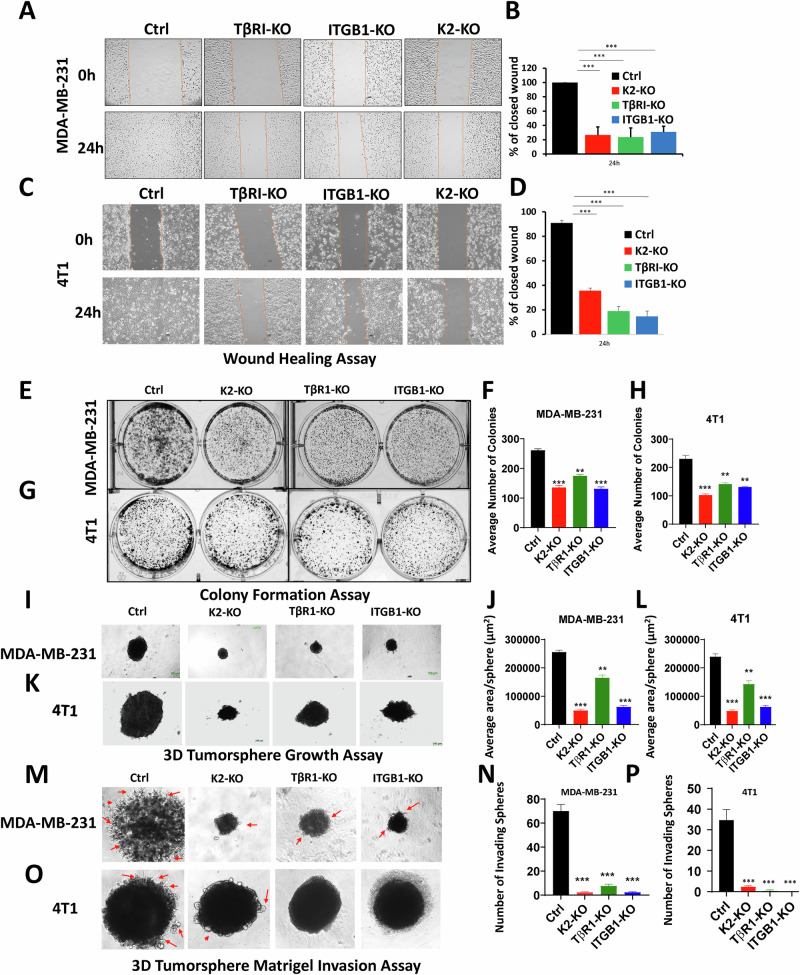
Loss of expression of either Kindlin-2, TβRI or β1-Integrin inhibits the in vitro oncogenic behavior of TNBC tumors. Wound healing assay (**A**–**D**), 2D-colony formation assay (**E**–**H**), 3D-tumorsphere growth assay (**I**–**L**), and 3D-tumorsphere invasion assay (**M**–**P**) of control (Ctrl) MDA-MB-231 and 4T1 cells and their K2-KO, TβRI-KO and *ITGB1*-KO derivatives. Scale bar: 100 µm. Data are the mean ± SD (*n* = 3, **p < 0.01; ***p < 0.001, Student’s *t*-test).

**Fig. 4:**
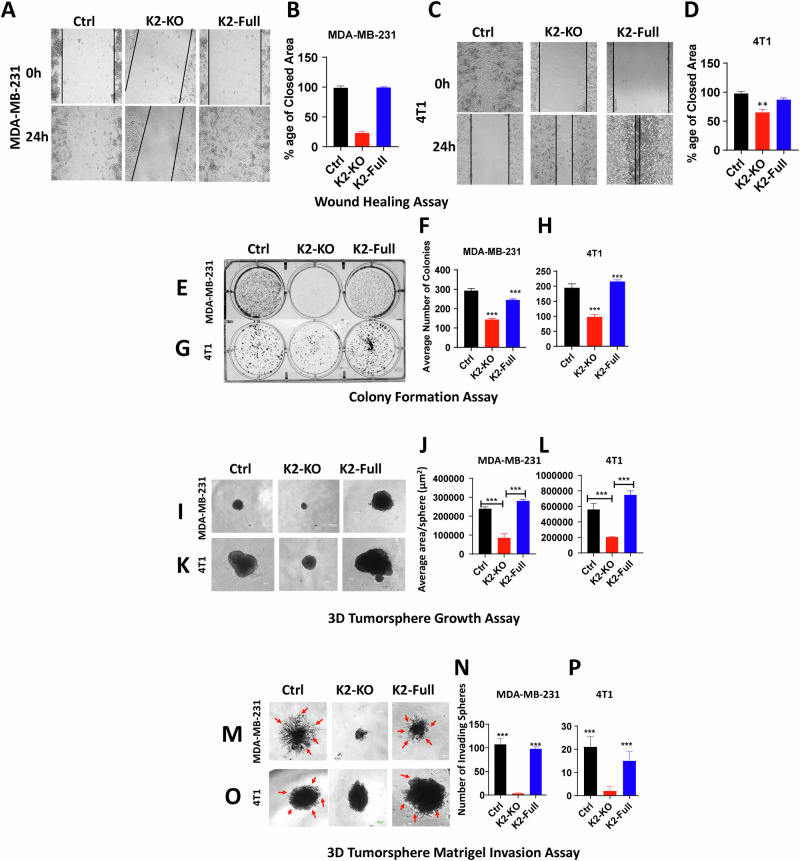
Re-expression of Kindlin-2 expression rescues the in vitro oncogenic behavior of TNBC tumors. Wound healing assay (**A**–**D**), 2D-colony formation assay (**E**–**H**), 3D-tumorsphere growth assay (**I**–**L**), and 3D-tumorsphere invasion assay (**M**–**P**) of control (Ctrl) MDA-MB-231 and 4T1 cells, and their K2-KO or K2-KO rescued with K2-full derivatives. Scale bar: 100 µm. Data are the mean ± SD (*n* = 3, **p < 0.01; ***p < 0.001, Student’s *t*-test).

To investigate the biological significance of loss of expression of either Kindlin-2, TβRI or β1-Integrin (*ITGB1*), and their effect on the oncogenic behavior of TNBC cells, we performed several in vitro assays. Parental MDA-MB-231 or 4T1, or their Kindlin-2-, *TβRI*- or *ITGB1*-deficient (KO) derivatives were subjected to the wound healing assay (Fig. 3A–D). Loss of expression of either Kindlin-2, TβRI or ITGB1 resulted in the inability of the MDA-MB-231-KO cells (Fig. 3A, B) or the 4T1 KO cells (Fig. 3C, D) to effectively close the scratch wound after 24 h, supporting the role of Kindlin-2, TβRI and β1-Integrin in cancer cell migration. Loss of expression of either Kindlin-2, TβRI or ITGB1 also inhibited the colony formation potential, a hallmark of cancer cells phenotype, of both MDA-MB-231 (Fig. 3E, F) and 4T1 (Fig. 3H) cells. In addition, to mimic the behavior of cancer cells in the tumor microenvironment, we performed 3D-tumorsphere growth and tumorsphere invasion of extracellular matrices (ECMs). 3D-tumorsphere growth was significantly (*p* < 0.01) inhibited in both the MDA-MB-231 (Fig. 3I, J and Fig. S2A) and the 4T1 (Fig. 3, L and Fig. S2B) cells deficient in either Kindlin-2, β1-Integrin or ITGB1 K2-KO. Similarly, MDA-MB-231 or 4T1 cells (Fig. 3M, N and Fig. 3O, P, respectively) were unable to invade ECM containing Matrigel (Fig. 3M–P, and Fig. S3A, B). Interestingly re-expression of full-length Kindlin-2 (K2-Full) in K2-KO MDA-MB-231 or 4T1 cells restored the cell migration potential of MDA-MB-231 cells (Fig. 4A, B) and 4T1 cells (Fig. 4C, D). Colony formation activity was also restored in the K2-KO MDA-MB-231 and 4T1 cells re-expressing K2-Full (Fig. 4E, F and Fig. 4G, H, respectively). In a similar manner, the ability of MDA-MB-231 and 4T1 cells to establish tumorspheres in 3D organoid growth assays (Fig. 4I–L, and Fig. S4A, B) and for these tumorspheres to invade ECMs (Fig. 4M-P, and Fig. S5A, B) were also restored in the K2-KO MDA-MB-231 and 4T1 cells re-expressing full-length Kindlin-2. These data have so far demonstrated the requirement of Kindlin-2, TβRI and ITGB1 for the common oncogenic activities of cancer cells, and for Kindlin-2 to be sufficient to restore these activities downstream of either β1-Integrin and TβRI.

### Loss of expression of either Kindlin-2, TβRI or ITGB1 inhibits signaling activities that are specific to β1-Integrin and TβRI

**Fig. 5:**
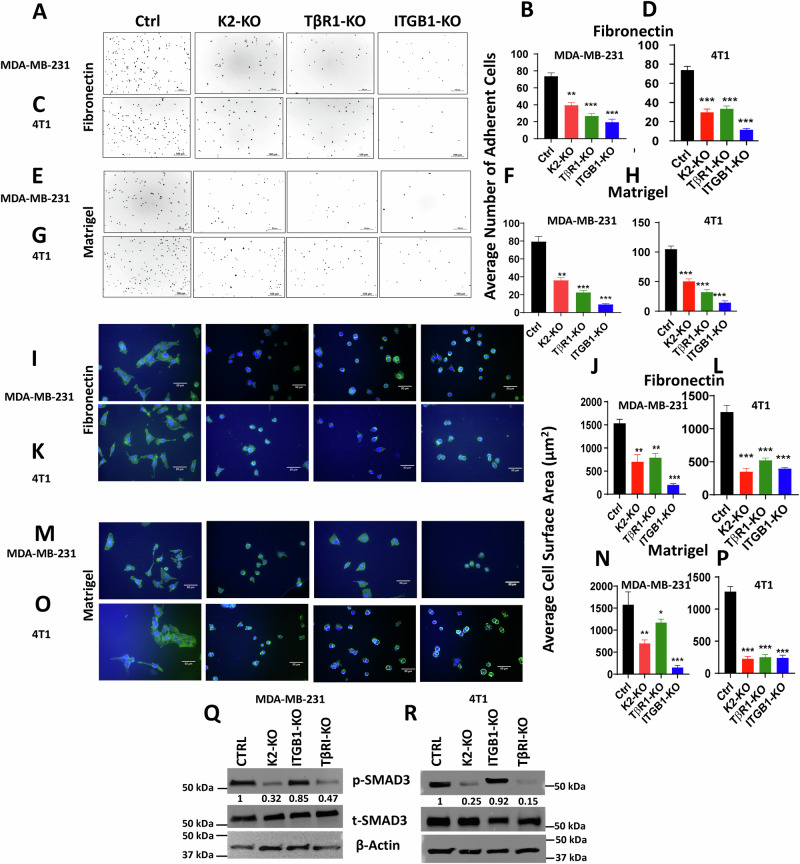
Loss of expression of either Kindlin-2, TβRI or ITGB1 inhibits signaling activities that are specific to β1-Integrin and TβRI. Representative images and average number of adherent control (Ctrl) MDA-MB-231 and 4T1 cells, and their K2-KO, TβRI-KO and ITGB1-KO derivatives on fibronectin (**A**–**D**), and on Matrigel (**E**–**H**). **I**–**N** Representative images and average cell surface area of control (Ctrl) MDA-MB-231 and 4T1 cells, and their K2-KO, TβRI-KO and ITGB1-KO derivatives on fibronectin (**I**–**L**), and on Matrigel (**M**–**P**) after spreading assay. Actin filaments were stained with Alexa 488 Phalloidin and nuclei were counterstained with DAPI. **Q**, **R**, WB results of phosphorylation levels of SMAD2/3 in control (Ctrl) MDA-MB-231 and 4T1 cells, and their K2-KO, TβRI-KO and ITGB1-KO derivatives. Scale bar: 100 µm for adhesion images and 50 µm for spreading images. The numbers under each WB band represent the fold change in signal intensity with respect to its respective control band in each panel after normalization to the loading control signal. Data shown are representative of 3 replicates. Data are the mean ± SD (*n* = 3, **p < 0.01; ***p < 0.001, Student’s *t*-test).

In the next set of experiments, we investigated the effect of loss of either Kindlin-2, TβRI or ITGB1 on oncogenic activities that are specifically activated downstream of either β1-Integrin or TβRI. For the β1-Integrin downstream activities, we assessed for cell adhesion on fibronectin, a cellular activity that is regulated by integrins [34] (Fig. 5A–D). Loss of expression of either of the three genes resulted in a significant (*p* < 0.001) inhibition of cell adhesion of MDA-MB-231 and 4T1 cells to Fibronectin (Fig. 5A, B and Fig. 5C, D, respectively). Adhesion of MDA-MB-231 and 4T1 cells to Matrigel (Fig. 5E–H, respectively) or Laminin (Fig. S6) was also significantly (*p* < 0.001) inhibited in the KO cells. Cell spreading on ECM is another major cellular activity that is tightly regulated by integrins [34]. Here again, we found loss of expression of either Kindlin-2, TβRI or ITGB1 resulted in a significant *(p* < 0.001) inhibition of spreading of MDA-MB-231 and 4T1 to Fibronectin (Fig. 5I–L, respectively), and to Matrigel (Fig. 5M, N and Fig. O&P, respectively), or to Laminin (Fig. S7). Thus we show loss of expression of either Kindlin-2, TβRI or ITGB1 to inhibits cellular activities that are specific to Integrins. Since β1-Integrin can form heterodimers with other α-Integrin subunits [35, 36], we used qt-RT-PCR to assess for expression levels of α1, αV and α5, which are predominantly expressed in cancer cells [37]. Loss of Kindlin-2 did not affect expression levels of either α1, αV and α5 subunits (Fig. S8A). However, loss of either ITGB1 or TβRI resulted in a significant (p < 0.01) inhibition of expression of both αV and α5 subunits, but not those of α1 subunit (Fig. S8B and S8C, respectively). This is consistent with the spreading data obtained on Fibronectin and Laminin since both αV and α5 integrin subunits, but not α1 subunit are involved in the β1-Integrin-mediated interaction with Fibronectin and Laminin [36]. Finally, as a readout for the molecular signaling downstream of TβRI, we assessed for phosphorylation levels of SMAD2/3 that takes place downstream of the TGF-β-mediated activation of the TβRI:TβRII complex. Loss of expression of either of the three proteins also resulted in reduction in phosphorylation levels of SMAD2/3 in both MDA-MB-231 (Fig. 5Q) and 4T1 cells (Fig. 5R). Thus, we show that loss of expression of either Kindlin-2, TβRI or ITGB1 inhibited the TβRI-specific downstream signaling, and, therefore, implicating Kindlin-2 as a major player in the regulation of the downstream signaling effectors of both β1-Integrin and TβRI.

### Re-expression of Kindlin-2 in the K2-deficient TNBC cells is sufficient for the restoration of the oncogenic activities downstream of β1-Integrin and TβRI

**Fig. 6:**
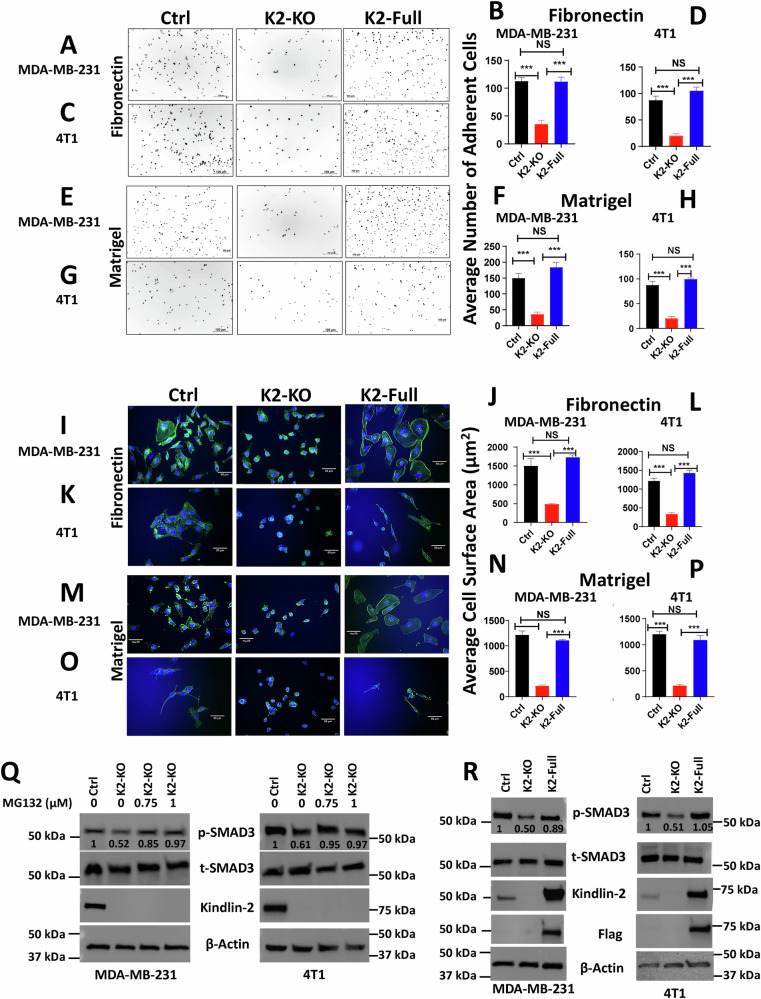
Re-expression of Kindlin-2 in the K2-deficient TNBC cells is sufficient for the restoration of the oncogenic activities downstream of β1-Integrin and TβRI. Representative images and average number of adherent MDA-MB-231 and 4T1 cells, and their K2-KO and K2-KO rescued with K2-full derivatives on Fibronectin (**A**–**D**), and on Matrigel (**E**–**H**). **I**–**P** Representative images and average cell surface area of MDA-MB-231 and 4T1 cells and their K2-KO and K2-KO rescued with K2-full derivatives after spreading assay on fibronectin (**I–L**) and on Matrigel (**M**–**P**). Actin filaments were stained with Alexa 488 Phalloidin and nuclei were counterstained with DAPI. WB analyses of phosphorylation od SMAD2/3 of control (Ctrl) MDA-MB-231 and 4T1 cells and their K2-KO derivatives after treatment with MG132 (**Q**) or after re-expression of full length in the K2-KO cells (**R**). Scale bar: 100 µm for adhesion images and 50 µm for spreading images. The numbers under each WB band represent the fold change in signal intensity with respect to its respective control band in each panel after normalization to the loading control signal. Data shown are representative of 3 replicates. Data are the mean ± SD (*n* = 3, ***p < 0.001, ANOVA).

To determine whether the cellular activities that are regulated downstream of β1-Integrin are mediated by Kindlin-2, the K2-KO MDA-MB-231 and 4T1 TNBC cells re-expressing full-length Kindlin-2 were subjected to cell adhesion and spreading. Cell adhesion on fibronectin was fully restored in both K2-deficient MDA-MB-231 cells (Fig. 6A, B) and 4T1 cells (Fig. 6C, D) re-expressing Kindlin-2. Cell adhesion on Matrigel was also fully restored for both cell lines (Fig. 6E, F, and Fig. 6G, H), as well as Laminin (Fig. S9). Similarly, re-expression of full-length Kindlin-2 in the K2-KO cells also fully restored the spreading potential of both cell lines on fibronectin (Fig. 6I–L for MDA-MB-231 and 4T1, respectively), Matrigel (Fig. 6M–P for MDA-MB-231 and 4T1), and Laminin (Fig. S10). Phosphorylation levels of SMAD2/3, a readout of TβRI-specific downstream signaling activity, was also restored in K2-KO MDA-MB-231 cells treated with the proteosome inhibitor MG132 (Fig. 6Q), as well as in the K2-KO cells re-expressing full-length Kindlin-2 (Fig. 6R). Thus we confirm that the signaling activities that are mediated downstream of β1-Integrin or TβRI are specifically regulated by Kindlin-2. Additionally, we also confirmed that Kindlin-2 expression, by stabilizing the β1-Integrin:TβRI protein complex is sufficient for the restoration of these β1-Integrin and TβRI downstream cellular and signaling activities.

### Loss of expression of either Kindlin-2, TβRI or ITGB1 inhibits growth and metastasis of TNBC tumors, which can be restored by re-expression of Kindlin-2

**Fig. 7:**
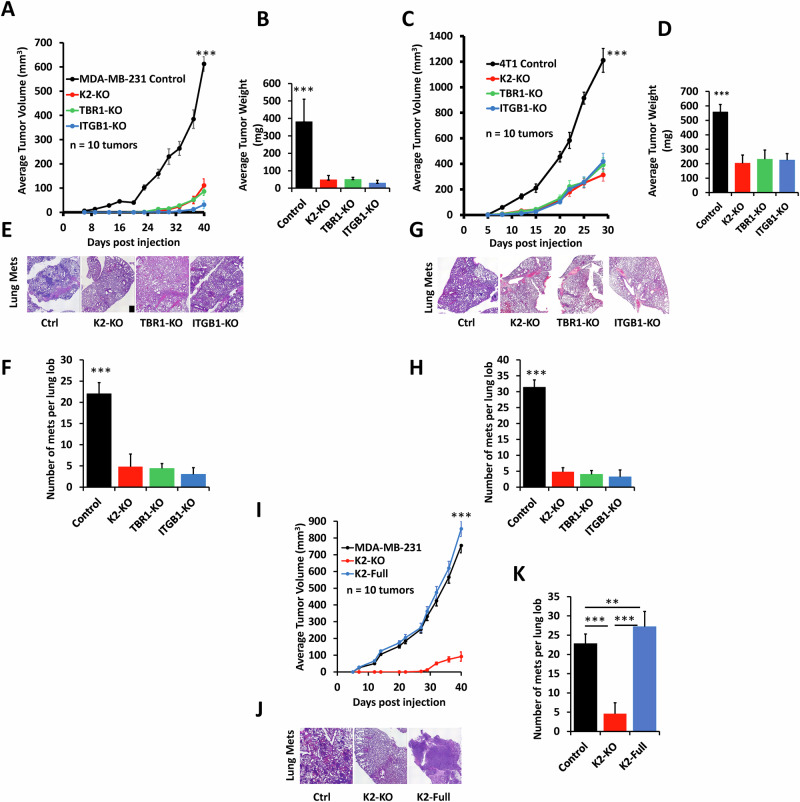
Loss of expression of either Kindlin-2, TβRI or ITGB1 inhibits growth and metastasis of TNBC tumors, which can be restored by re-expression of Kindlin-2. Tumor volume (**A**, **C**) and weight (**B**, **D**) of NSG mice (**A**, **B**) or Balb/C mice (**C**, **D**) injected with control (Ctrl) MDA-MB-231 or 4T1 cells or their K2-KO, TβRI-KO and ITGB1-KO derivatives up to 40 days post injection. (Representative H&E staining (**E**, **G**) of lungs form tumor-bearing mice from **A** and **C**, and quantification of metastatic foci (**F**, **H**). **I** Tumor volume of NSG mice injected with control (Ctrl) MDA-MB-231 cells, and their K2-KO or K2-KO rescued with K2-full derivatives up to 36 days post injection. **J** Representative H&E staining of their corresponding lungs, and quantification of metastatic foci (**K**). **p < 0.01; ***p < 0.001; ANOVA.

Next, we determined whether the biological and signaling effects observed in vitro, can also be recapitulated in vivo in mouse models for TNBC tumor progression and metastasis. Using the spontaneous metastasis mouse model, MDA-MB-231 or 4T1 TNBC control cells and their KO derivatives were injected in the mammary fat pads of NSG mice (MDA-MB-231) or Balb/C mice (4T1), and growth of the primary tumors was monitored over time. Loss of expression of either of the three proteins resulted in a significant (*p* < 0.001) delay in tumor growth and weight in both the MDA-MB-231 model (Fig. 7A, B) as well as the 4T1 model (Fig. 7C, D). Metastasis was also significantly (*p* < 0.001) inhibited in the lungs of mice injected with the KO MDA-MB-231 cells (Fig. 7E, F) or with KO 4T1 cells (Fig. G&H). Re-expression of Kindlin-2 in the Kindlin-2-deficient MDA-MB-231 cells resulted in the rescue of both tumor growth (Fig. 7I) and metastasis (Fig. 7J, K). These data show the impact of Kindlin-2, TβRI and ITGB1 on tumor progression and metastasis. They also confirm the specificity of Kindlin-2 in this process, where Kindlin-2 is sufficient for the restoration of the growth and metastasis potentials of TNBC tumors that lack expression of either TβRI or ITGB1.

### Re-expression of Kindlin-2 lacking domains involved in the interaction of Kindlin-2 with β1-Integrin or TβRI does not fully restore the oncogenic behavior of TNBC tumors

**Fig. 8:**
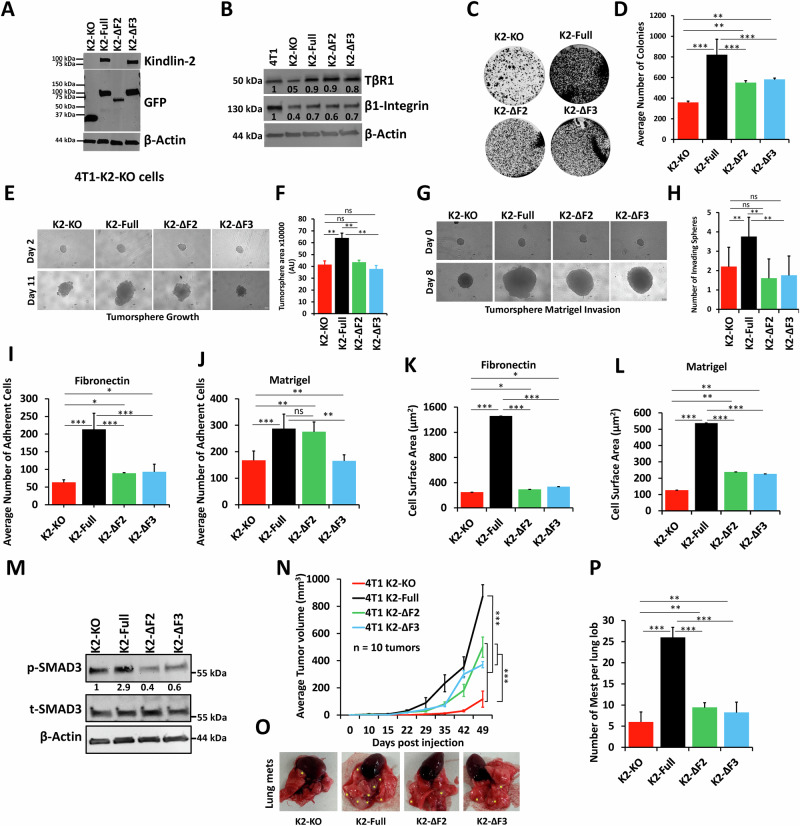
Re-expression of Kindlin-2 lacking domains involved in the interaction of Kindlin-2 with β1-Integrin or TβRI does not fully restore the oncogenic behavior of TNBC tumors. **A** Western Blot analysis with either Kindlin-2 or GFP antibodies showing expression of Kindlin-2-fusion derivatives from protein lysates of 4T1-K2-KO cells expressing either GFP, K2-Full-GFP, K2-ΔF2-GFP or K2-ΔF3-GFP. Note that the K2-ΔF2-GFP band is not detected by the Kindlin-2 antibody, since this antibody was raised against an epitope that resides within the F2 domain. **B** Western Blot analysis with indicated antibodies showing expressions of TβRI and β1-Integrin proteins from protein lysates of cells described in (**A**). β-Actin is a loading control in both A and B. 2D-colony formation assay (**C**, **D**), 3D-tumorsphere growth assay (**E**, **F**), and 3D-tumorsphere invasion assay (**G**, **H**) of 4T1-K2-KO cells, and their K2-Full, K2-ΔF2 or K2-ΔF3 rescued derivatives. Average number of adherent 4T1-K2-KO cells, and their K2-Full, K2-ΔF2 or K2-ΔF3 rescued derivatives on fibronectin (**I**), and Matrigel (**J**). **K**, **L** Average cell surface area of 4T1-K2-KO cells, and their K2-Full, K2-ΔF2 or K2-ΔF3 rescued derivatives. **I**, **J** Average number of spread 4T1-K2-KO cells, and their K2-Full, K2-ΔF2 or K2-ΔF3 rescued derivatives on fibronectin (**K**), and Matrigel (**L**). Scale bar: 100 µm for adhesion images and 50 µm for spreading images. **M** WB results of phosphorylation levels of SMAD2/3 4T1-K2-KO cells, and their K2-Full, K2-ΔF2 or K2-ΔF3 rescued derivatives. The numbers under each WB band represent the fold change in signal intensity with respect to its respective control band in each panel after normalization to the loading control signal. **N** Tumor volume of Balb/C mice injected with 4T1-K2-KO cells, and their K2-Full, K2-ΔF2 or K2-ΔF3 rescued derivatives up to 49 days post injection. Representative images of lungs (**O**) and quantification of metastatic foci (**P**) from mice described in (**N**). **p < 0.01; ***p < 0.001; ANOVA.

Finally we asked whether re-introducing Kindlin-2 deletion variants that lack either the F2 or the F3 domain, that are required for the interaction with TβRI and β1-Integrin, respectively, would affect the oncogenic activities downstream of TβRI and/or β1-Integrin. We generated K2-KO-4T1 cell variants to express either GFP alone, K2-Full-GFP, ΔF2-K2-GFP, or ΔF3-K2-GFP (Fig. 8A). Western Blot analyses showed that re-expression of full-length Kindlin-2 in the K2-KO 4T1 cells to partially restore expression of both TβRI and β1-Integrin, as did the ΔF2, and ΔF3 Kindlin-2 variants (Fig. 8B). We also found that the re-expression of full-length Kindlin-2 partially restored colony formation (Fig. 8C, D), tumorsphere growth (Fig. 8E, F) and tumorsphere invasion (Fig. 8G, H), while re-expression of either ΔF2-K2 or ΔF3-K2 failed to restore these oncogenic activities since they remained close to those achieved in K2-KO group. Thus, these findings further support the requirement of these two domains, and therefore, the interaction of K2 with TβRI and β1-Integrin to maintain the oncogenic behavior of TNBC cells in vitro.

Next, we assessed for the effect of re-expression of ΔF2-K2 or ΔF3-K2 on the oncogenic activities downstream of either TβRI or β1-Integrin. Here again, we found that while K2-Full was able to restore the signaling activities downstream of β1-Integrin by restoring cell adhesion (Figs. 8I, J and S11) and cell spreading (Figs. 8K, L and S12) on different extracellular matrices, while neither ΔF2-K2 nor ΔF3-K2 were able to do so. Similarly, K2-Full was able to restore the signal transduction downstream of TβRI by increasing pSMAD expression levels as compared to K2-KO (Fig. 8M), while neither ΔF2-K2 nor ΔF3-K2 did. Thus, both the F2 and F3 domains, that are involved in the interaction of Kindlin-2 with TβRI and β1-Integrin, respectively, are required for the oncogenic activities of TNBC cells downstream of both TβRI and β1-Integrin.

Finally, we assessed for the effect of ΔF2-K2 and ΔF3-K2 on tumor growth and metastasis, and found that mice injected with K2-full-expressing K2-KO-4T1 cells showed tumor growth that is significantly (*p* > 0.001) higher than that in mice injected with K2-KO-4T1 cells (Fig. 8N). On the other hand, the tumors derived from mice injected with cells expressing either ΔF2-K2 and ΔF3-K2, although noticeably bigger than those derived from K2-KO cells, they were still significantly (*p* < 0.001) smaller than those derived from cells expressing full-length K2 (Fig. 8N). Metastatic dissemination to the lungs was also fully restored in mice injected with K2-Full-expressing K2-KO-4T1 cells, while ΔF2-K2- or ΔF3-K2-expressing K2-KO-4T1 cells only resulted in a partial restoration of the metastatic potential of 4T1 cells (Fig. 8O, P). These results suggest that, while the F2 and F3 domains of Kindlin-2 are required for full restoration of tumor growth and metastasis, their deletion can still lead to a partial activation of the invasion-metastasis cascade of TNBC tumors. Together, our data support an important role for Kindlin-2 in the regulation of key oncogenic activities downstream of TβRI and β1-Integrin, both in vitro and in vivo.

## Discussion

Kindlin-2 initially garnered attention for its pivotal role in activating integrins, thereby mediating cell-extracellular matrix adhesion and signaling [23, 38–43]. This function is crucial for facilitating the interaction between cells and their extracellular environment by modulating integrin activity [23, 38–43]. Integrins, as transmembrane receptors, play a key role in cell adhesion and signal transduction. Kindlin-2 binds to the cytoplasmic tails of integrins, promoting their activation and enabling interaction with extracellular ligands. This activation is vital for cell adhesion, migration, and communication with the surrounding microenvironment. The dynamic interplay between Kindlin-2 and integrins significantly contributes to cell behavior under both normal physiological conditions and pathological manifestations such as cancer. In normal cellular functions, this relationship is crucial for embryonic development, tissue homeostasis, and immune responses [14]. However, dysregulation of Kindlin-2/integrin interactions has been linked to various diseases, particularly cancer, where aberrant cell adhesion and migration are prominent features [12].

Beyond its role in integrin activation, Kindlin-2 has emerged as a key player in regulating transforming growth factor-beta (TGF-β) signaling, the activating ligand of the TβRI:TβRII signaling complex [28, 44]. This interplay between Kindlin-2 and TGF-β regulates various cellular processes and contributes to both normal development and pathological conditions, including BC pathology [15]. The Kindlin-2-TGF-β axis plays a crucial role in epithelial-mesenchymal transition (EMT), a process central to embryonic development, while also implicated in cancer metastasis [16, 45]. Dysregulation of TGF-β signaling is a hallmark of cancer progression, and Kindlin-2 has been implicated in mediating TGF-β effects on tumor cell behavior [15, 16]. Notably, the interaction between Kindlin-2 and TGF-β receptors enhances cellular responsiveness to TGF-β and its downstream signaling, influencing processes such as cell proliferation and differentiation [28, 46].

Previously, our studies demonstrated that Kindlin-2 activates the CSF1/EGF paracrine oncogenic loop in BC through the regulation of TGF-β signaling [15]. Additionally, a study by Wei et al. [28] revealed Kindlin-2’s binding to the cytoplasmic region of TβRI. Consequently, Kindlin-2 plays a major role in regulating TNBC tumor progression and metastasis through the modulation of the oncogenic activities of both integrins and TGF-β.

In this paper, we present, for the first time and to the best of our knowledge, evidence of direct binding of Kindlin-2 to TβRI and β1-Integrin, whereby Kindlin-2 establishes a physical bridge between β1-Integrin and TβRI. Moreover, Kindlin-2, not only is necessary for the stabilization of the β1-Integrin:Kindlin-2:TβRI complex, but is also required to maintain the oncogenic behavior of TNBC cell lines both in vitro and in vivo. Indeed, Kindlin-2 is a crucial protein involved in integrin-mediated adhesion and signaling processes, essential for cell-extracellular matrix adhesion [47]. Kindlin-2 is believed to associate with β1-Integrin at nascent adhesions before talin recruitment during adhesion maturation, indicating its early involvement in the adhesion process [48] (Figs. S13 and S14). Disrupting the interaction between Kindlin-2 and integrin inhibits adhesion formation, integrin activation, and cell spreading, underscoring the significance of Kindlin-2 in these processes [31, 49–51].

Conversely, TβRI plays a vital role in the TGF-β/SMAD signaling pathway, regulating cell growth, differentiation, and migration, making it a central mediator of cancer progression [52]. Studies on TβRI and SMAD in BC emphasize the multifaceted role of the TGF-β signaling pathway and its components in the disease. While genetic variants such as TβRI*6A have shown associations with BC risk and progression, the regulatory mechanisms and functional implications of TβRI and SMAD signaling in BC require further investigation for a comprehensive understanding of their potential as therapeutic targets. Our study demonstrates that the loss of expression of Kindlin-2, TβRI, or β1-Integrin proteins results in reduction in phosphorylation levels of SMAD2/3 in both MDA-MB-231 and 4T1 cells. Activated TβRI phosphorylates SMAD2 and SMAD3, which heterodimerize with SMAD4 and translocate to the nucleus, binding to DNA and regulating the transcription of target genes involved in various cellular functions [53]. A recent study has also reported on the interplay between β1-Integrin, Kindlin-2, and the TβRI partner, TβRII, to promote pancreatic tumor growth [46]. This study establishes the molecular mechanism by which the interplay between these oncogenic proteins is regulated in the context of BC progression and metastasis.

Our investigation employed a combination of in vitro assays, two different mouse models for TNBC tumors, as well as genetic and pharmacological manipulation to establish Kindlin-2’s role as a major contributor to the stabilization of the TβRI:β1-Integrin protein complexes and the regulation of their downstream oncogenic activities, driving the progression and metastasis of TNBC tumors. We show that Kindlin-2 establishes direct interactions with TβRI and β1-Integrin, with the interaction between Kindlin-2 and TβRI mediated through the F2 domain of Kindlin-2 and the interaction between Kindlin-2 and β1-Integrin-mediated through the F3 domain of Kindlin-2 via a QW amino acid doublet. CRISPR/Cas9-mediated knockout of Kindlin-2 leads to the loss of both TβRI and β1-Integrin proteins, resulting in the destabilization of the protein complex, which can be rescued by inhibiting the proteasome degradation machinery or re-expressing full-length Kindlin-2 in Kindlin-2-deficient cells. This supports the novel function of Kindlin-2 in establishing a physical bridge between TβRI and β1-Integrin and the requirement of Kindlin-2 for the stabilization of this complex. Loss of expression of either Kindlin-2, TβRI, or β1-Integrin leads to the inhibition of in vitro and in vivo oncogenic behavior of TNBC cells, highlighting the importance of either member of this protein complex in maintaining the oncogenic behavior of cancer cells; loss of one member of this complex is sufficient for the mitigation of the oncogenic activities of cancer cells. The downstream signaling effectors specific to either TβRI (phosphorylation of SMAD2/3) or β1-Integrin (cell adhesion and spreading on fibronectin) can be inhibited by simply losing Kindlin-2, emphasizing the specificity of Kindlin-2 in modulating TβRI- and β1-Integrin-mediated regulation of the oncogenic behavior of cancer cells.

**Fig. 9:**
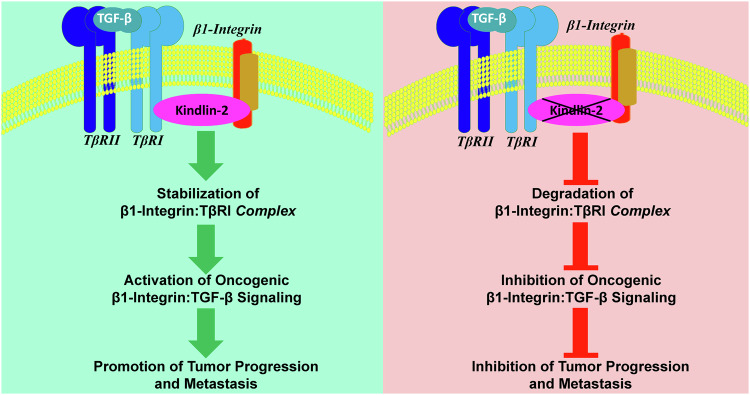
Model depicting the role of Kindlin-2 as a physical bridge maintaining the stability of the β1-Integrin:K2:TβRI protein complexes and their effect on the regulation of the downstream oncogenic behavior of TNBC tumors.

Our study unveils, for the first time, the intricate relationship between Kindlin-2, integrins, and TβRI, regulating crucial cellular processes (Fig. 9). This trilateral interplay integrates the roles of focal adhesion protein Kindlin-2, transmembrane receptors integrins, and TβRI in orchestrating various physiological and pathological events. At the core of this relationship lies the modulation of integrin activation by Kindlin-2. Kindlin-2 interacts with the cytoplasmic tails of integrins, promoting their activation and enabling their interaction with extracellular ligands. This activation is essential for cellular adhesion and sets the stage for downstream signaling events. On the other hand, Kindlin-2 influences the TGF-β signaling pathway through its interaction with TβRI, enhancing its activation and promoting downstream TGF-β signaling cascades. This interaction contributes to the regulation of cellular processes such as proliferation, differentiation, and migration, emphasizing the central role of Kindlin-2 in integrating signals from both integrins and TGF-β. Importantly, in clinical settings, dysregulation of this Kindlin-2:Integrins:TβRI axis has implications in diseases such as cancer, where aberrant integrin activation and TGF-β signaling are hallmarks of cancer progression, and Kindlin-2 emerges as a potential key player bridging these pathways. Finally, it is important to emphasize that Kindlin-2 may regulate other oncogenic signaling pathways, beyond its involvement in the stabilization of the β1-Integrin:TβRI protein complex. Analysis of RNA-seq data obtained from MDA-MB-231 cells and their K2-deficient derivatives [21] showed that, in addition to the regulation of EMT and ECM (Fig. S15), Kindlin-2 may also be involved in the regulation of a range of other oncogenic signaling pathways, such as KRAS, angiogenesis, immune evasion (Fig. S15), as wells as transmembrane signaling, cell motility, apoptosis, inflammatory response …etc. (Fig. S16). These findings and observations, strongly support the role of Kindlin-2 as a major player in the regulation of several hallmarks of cancer.

## Conclusions

In conclusion, the β1-integrin:Kindlin-2:TβRI interconnection represents a sophisticated network that regulates BC progression and metastasis. Further investigations of this trilateral oncogenic axis holds promise for deciphering the intricacies of cellular behavior in disease, and therefore offering potential therapeutic avenues to specifically target these pathways for the treatment of TNBC tumors and other tumors.

## Materials and methods

### Cell lines and reagents

MDA-MB-231, 4T1, and HEK293 cells were procured from the American Type Culture Collection (ATCC; Manassas, VA) and maintained in accordance with the manufacturer’s specified protocols. Although cell line authentication was not explicitly conducted, we relied on the manufacturer’s quality control assurances. Periodic testing for Mycoplasma contamination was performed every 9–12 months. All cells were cultured at early passages (no more than 15), and each culture was passaged no more than five times before introducing a fresh vial. Kindlin-2, TβRI, and ITGB1-deficient cells were generated through electroporation of cancer cells with a ribonucleoprotein mixture of guide RNAs (sgRNA) and Cas9 (Synthego), following the manufacturer’s instructions (Table S1). A pool of three verified sgRNAs was used for each human or mouse gene (Synthego), with scrambled sgRNAs serving as a negative control. Western blot (WB) analysis validated efficient and stable knockout (KO). In cases where knockdown efficiency was below 80%, a second round of sgRNA delivery was implemented. No antibiotic selection was required, as the knockdown efficiency was sustained throughout the cells’ utilization. Primary antibodies for TβRI, phospho-Smad2/3, Smad3 were obtained from Abcam, Inc. Mouse monoclonal anti-Kindlin-2, clone 3A3 was sourced from EMD Millipore. Rabbit anti-Integrin β1 and PE-conjugated anti-Integrin β1/CD29 (GeneTex), FITC-conjugated HUTS-4 antibody (Sigma). Rabbit polyclonal anti-FAK (Invitrogen), Mouse monoclonal anti-Vinculin (Sigma). Secondary antibodies for IF were donkey anti-mouse IgG Alexa 594 and Donkey anti-Rabbit IgG Alexa 488 (Invitrogen). Goat horseradish peroxidase-conjugated anti-mouse IgG and goat horseradish peroxidase-conjugated anti-rabbit IgG for western blot (Bio-Rad). Gel electrophoresis reagents for protein and DNA were from Bio-Rad.

### Co-Immunoprecipitation and western blotting

Cells were lysed with RIPA or NP40 lysis buffer with proteases and phosphatases inhibitor cocktails. Total protein quantification was performed using the BCA protein assay kit (Bio-Rad). Co-immunoprecipitation analysis involved incubating lysates at 4 °C with protein A resin and specific antibodies, as described previously [54]. Western blot assays followed standard protocols with β-Actin as the loading standard. The ChemiDoc MP Imaging system (Bio-Rad) was employed for image acquisition of developed membranes, and intensity of WB bands was quantified using ImageJ software.

### Solid phase binding assay

Full-length Kindlin-2 was expressed in Escherichia coli as GST fusion protein and purified on Glutathione-Sepharose (GE Healthcare) according to manufacturer’s instructions. Recombinant β1-Integrin (Abcam, Cat. # ab114157) or TβRI (Abcam, Cat. # ab105908) protein in TBS buffer was immobilized onto 96-well microtiter plates (Corning Costar Corp., Cambridge, MA) at 5 μg/well for 20 h at 4 °C. After post-coating with 3% BSA for 1 h at 37 °C, Kindlin-2-GST-tagged protein was added (0–7 µM) in TBS buffer containing 1 mM CaCl_2_, 1 mM MgCl_2_ and incubated for 2 h at 37 °C. GST and p53-GST proteins were used as negative controls. After 3 washings with TBS anti-GST-HRP Ab (1:2000) was added (100 µl/well) and incubated for 1 h at RT. The plates were washed 3 times with TBS, developed with 1-Step Ultra TMB-ELISA substrate (ThermoScientific) and absorbance at 450 nm was measured. In isotherm binding studies, input concentrations of the GST–Kindlin-2 required for half-maximal binding to immobilized β1-Integrin or TβRI proteins were estimated using the Sigma Plot software (SPSS) in which the data were fitted to a one-site binding equation. GST alone was as a negative control for binding and for the normalization. p-53-GST was used a second negative control for binding.

### Colony formation assay

MDA-MB-231 (3000 cells) and 4T1 (1000 cells) were seeded into 6-well plates. Cultured for 10 days, fresh medium was supplemented every 3 days. Clones were washed with PBS, fixed with 4% paraformaldehyde (PFA) at room temperature for 20 minutes, and stained with 0.25% crystal violet solution. Image acquisition of the 6-well plate was performed using the ChemiDoc MP Imaging system (Bio-Rad) and ImageJ software, and quantification of clones was done using ImageJ software.

### Wound healing assay

Cells were seeded in 6-well plates, grown to a confluent monolayer, and subjected to a scratch wound. After a quick wash with PBS, cells were cultured for 24 h. Images at 0 and 22 h. post-wounding were acquired using a Nikon ECLIPSE TS2r microscope, and the remaining open area was calculated using ImageJ software.

### RNA Extraction and quantitative real-time RT- PCR

Total RNA was isolated using TRIzol reagent (Invitrogen) and quantified with Nanodrop. Reverse transcription and quantitative real-time PCR (Bio-Rad) were performed using the High Capacity cDNA Reverse Transcription Kit (Invitrogen) and SYBR Green Master Mix Kit (Invitrogen), respectively. Primers were obtained from Qiagen (Table S2).

### 3D-tumorsphere growth and invasion assays

For 3D single-tumorsphere formation, cells were seeded into a round bottom 96-well ultralow attachment (ULA) plate and monitored for 11 days, as described previously [55]. Invasion assays involved supplementing tumorsphere cultures with Matrigel, and invasion was monitored for 8 additional days. Images were captured and quantified using ImageJ software, as described previously [55].

### Cell adhesion and spreading assays

Adhesion and spreading assays were performed as described previously [55]. Cells were seeded onto coverslips precoated with fibronectin, laminin, and Matrigel. Actin filaments were stained with Alexa 488 Phalloidin and nuclei were counterstained with DAPI. Adhered cells were imaged, and spreading was assessed by capturing different fields and quantifying the area around the cells using ImageJ software.

### Flow cytometry analyses

Cell surface expression and activation of β1-Integrin was assessed by FACS (Sony ID7000), as described previously [55]. Cells were detached using trypsin, washed, and resuspended for staining with PE-conjugated mouse anti-human CD-29 antibody or FITC-conjugated HUTS-4 antibody. Data were analyzed using FlowJo software.

### Immunofluorescence and confocal microscopy

Cells were seeded on glass coverslips, processed, and incubated with primary antibodies overnight. After blocking and incubating with secondary antibodies, cells were mounted with DAPI-containing mounting medium. Images were captured on a LEICA DM5500 laser scanning confocal microscope.

### In vivo tumor growth and metastasis study

NSG female mice and BALB/C mice were purchased from Jackson and used for tumor growth and metastasis studies as described in our published studies [29, 56–58]. Parental and derivative cells were injected into mammary fat pads, in both the right and left sides (5 mice per group, 10 tumors per group), and tumor growth was monitored. Mice were sacrificed when to maximum tumor burden was reached in the control group, and tumors and lung metastases were analyzed as described [29, 56–59].

### Statistical analyses

Statistical analyses were performed using GraphPad Prism (version 8.0) and SPSS (version 21.0). All experiments were conducted in triplicate, and variables were expressed as mean ± SD. Student’s *t*-test was used, and significance was considered at *p* < 0.05. ANOVA was used when comparing more than two groups with continuous variables, and significance was considered at *p* < 0.05.

## Supplementary information

Suplementary figures and tables

## Data Availability

All data are contained within the article. Requests for reagents should be addressed to K Sossey-Alaoui (kxs586@case.edu).
