# Supplementary material for: Kindlin-2 regulates the oncogenic activities of integrins and TGF-β in triple-negative breast cancer progression and metastasis
Source: Oncogene. 2024 Sep 19;43(45):3291–305. doi: 10.1038/s41388-024-03166-2 (PMC11534691; doi:10.1038/s41388-024-03166-2)
Supplement: Supplementary file 1 — Suplementary figures and tables [file 41388_2024_3166_MOESM1_ESM.pdf]

Supplementary Materials for

Kindlin-2 Regulates the Oncogenic Activities of Integrins and TGF-β In Triple Negative Breast Cancer Progression and Metastasis

Neelum Aziz Yousafzai, Lamyae El Khalki, Wei Wang, Justin Szpendyk, Khalid Sossey-Alaoui.

Correspondence to: KXS586@Case.edu

This PDF file includes:

Figures. S1 to S16
Tables S1 to S2

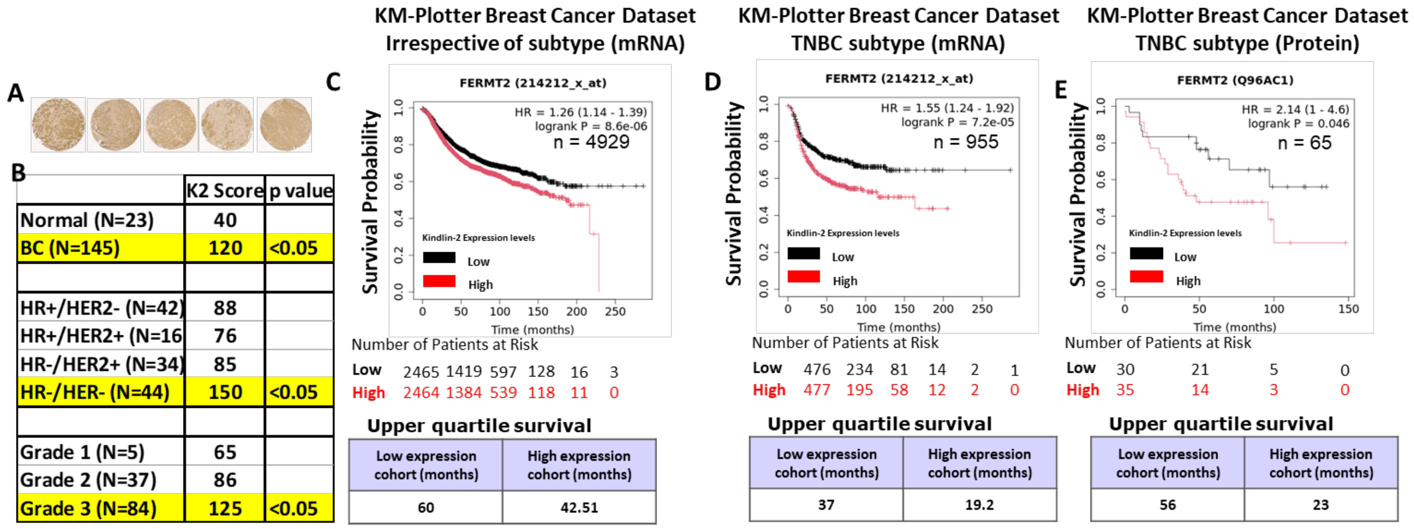

Figure. S1.

(A) Representative K2 immunostainings of advanced human BC tumors from a 150 BC TMA cohort. **(B)** Kindlin-2 IHC staining scores from panel A. **(C)** KM plot correlating survival of 3954 BC patients with K2 mRNA expression levels. **(D)** KM plot correlating survival of 955 TNBC patients with K2 mRNA expression levels. **(E)** KM plot correlating survival of 65 TNBC patients with K2 protein expression levels.

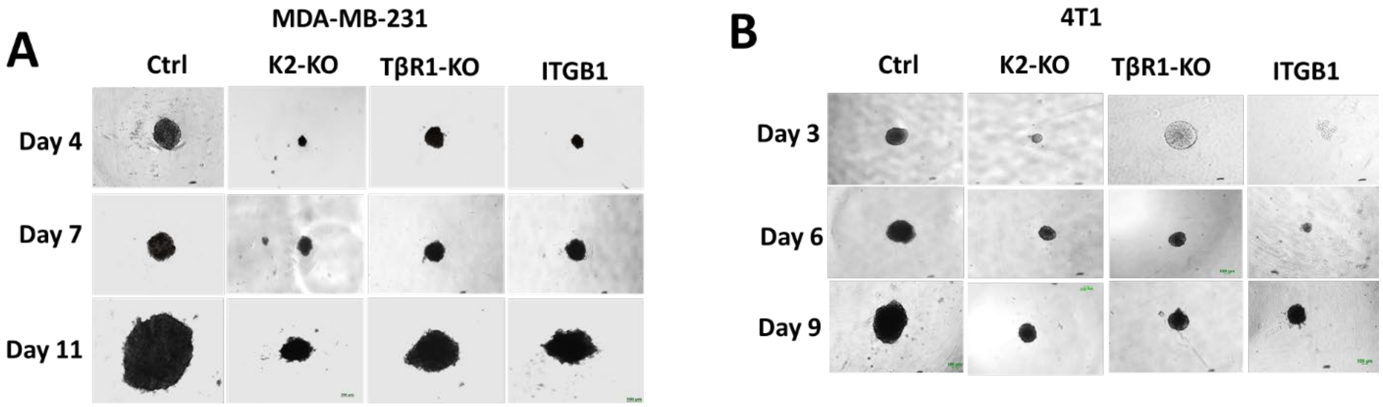

Figure. S2.

Representative pictograms of tumorspheres of MDA-MB-231 cells (A) and 4T1 cells (B) and their K2-KO, TβR1-KO, ITGB1-KO derivatives grown in 3D conditions. Scale bar :100 µm.

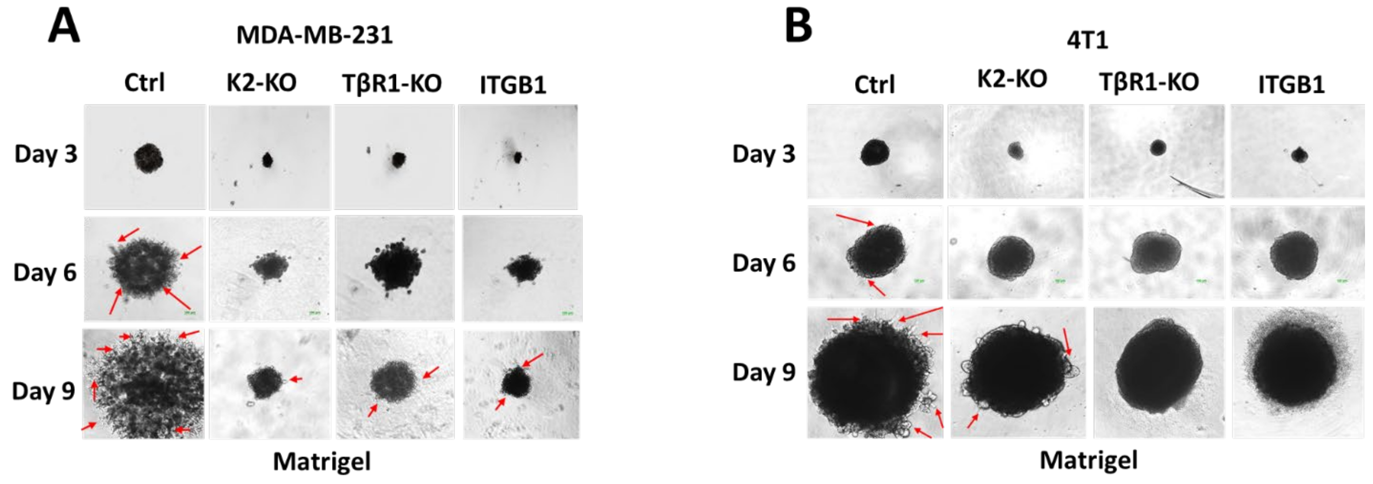

Figure. S3.

Representative pictograms of invasive tumorspheres of MDA-MB-231 cells (A) and 4T1 cells (B) and their K2-KO, TβR1-KO, ITGB1-KO derivatives grown in 3D conditions supplemented with Matrigel at day 3 after seeding. Red arrows point cells or cell groups that have invaded Matrigel beyond the primary tumorsphere. Scale bar :100 µm.

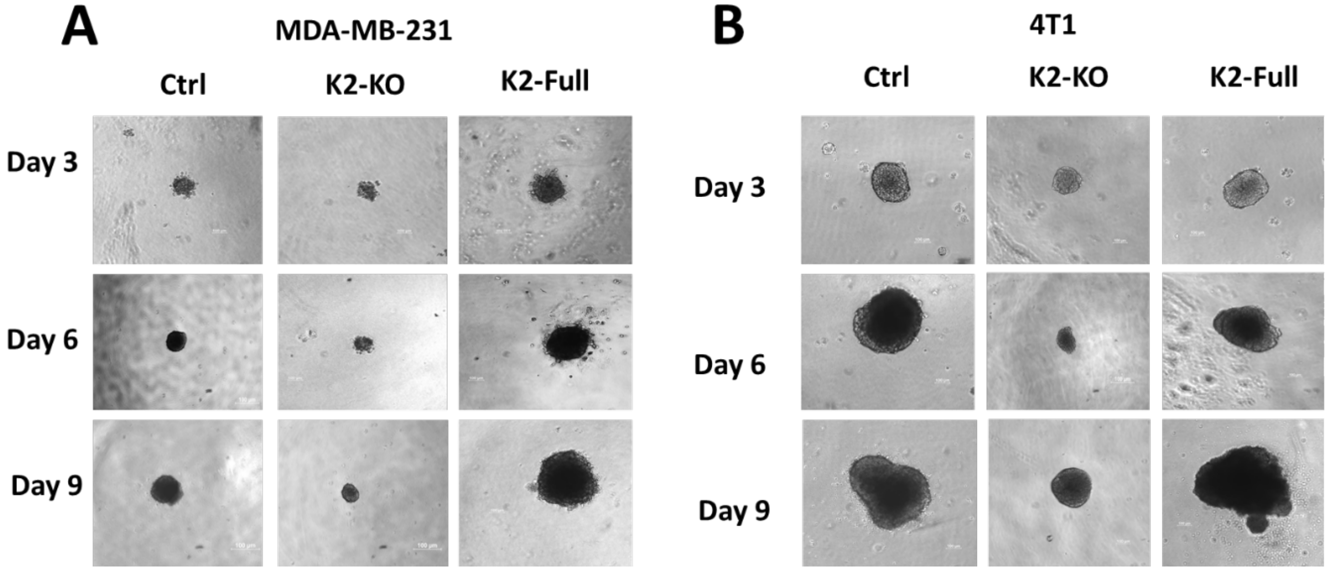

Figure. S4.

Representative pictograms of tumorspheres of MDA-MB-231 cells (A) and 4T1 cells (B) and their K2-KO and rescued K2-full derivatives grown in 3D conditions. Scale bar :100 µm.

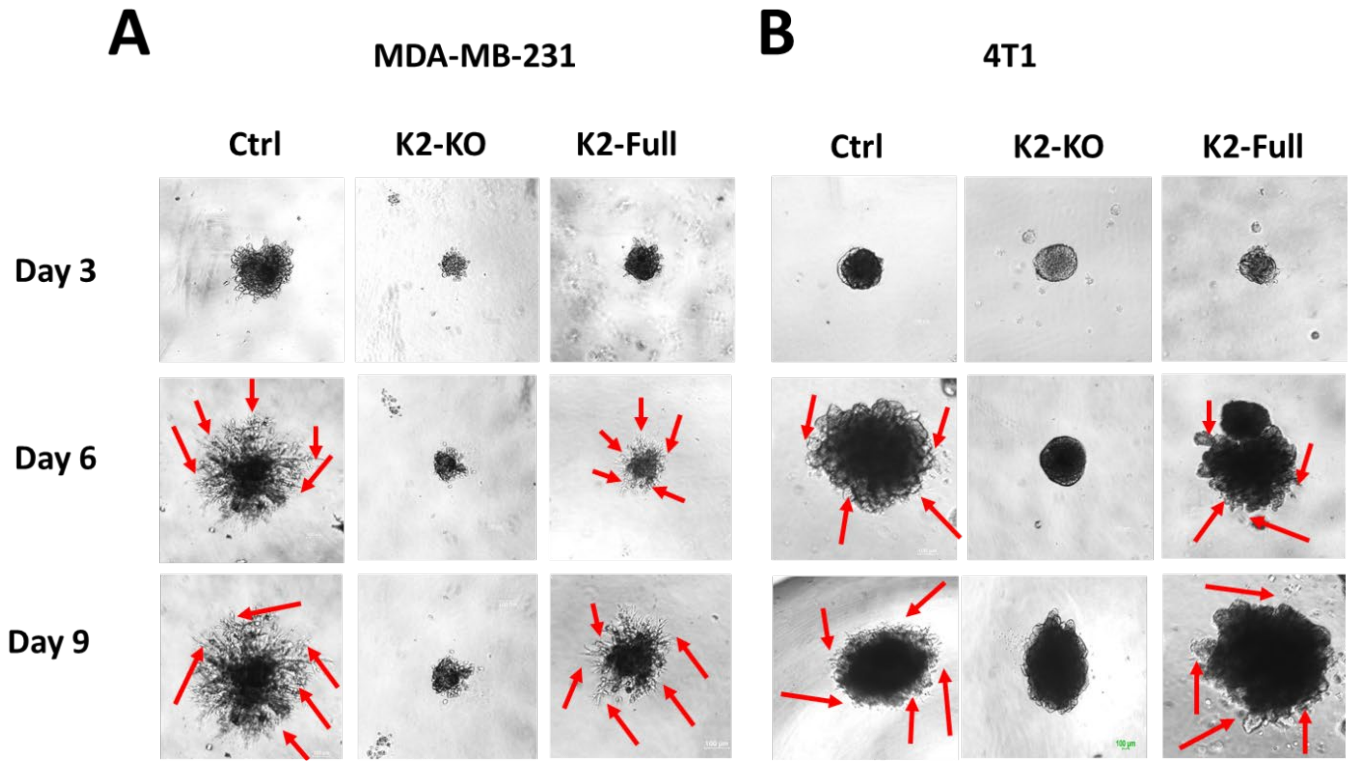

Figure. S5.

Representative pictograms of invasive tumorspheres of MDA-MB-231 cells (A) and 4T1 cells (B) and their K2-KO and rescued K2-full derivatives grown in 3D conditions supplemented with Matrigel at day 3 after seeding. Red arrows point cells or cell groups that have invaded Matrigel beyond the primary tumorsphere. Scale bar :100 μm.

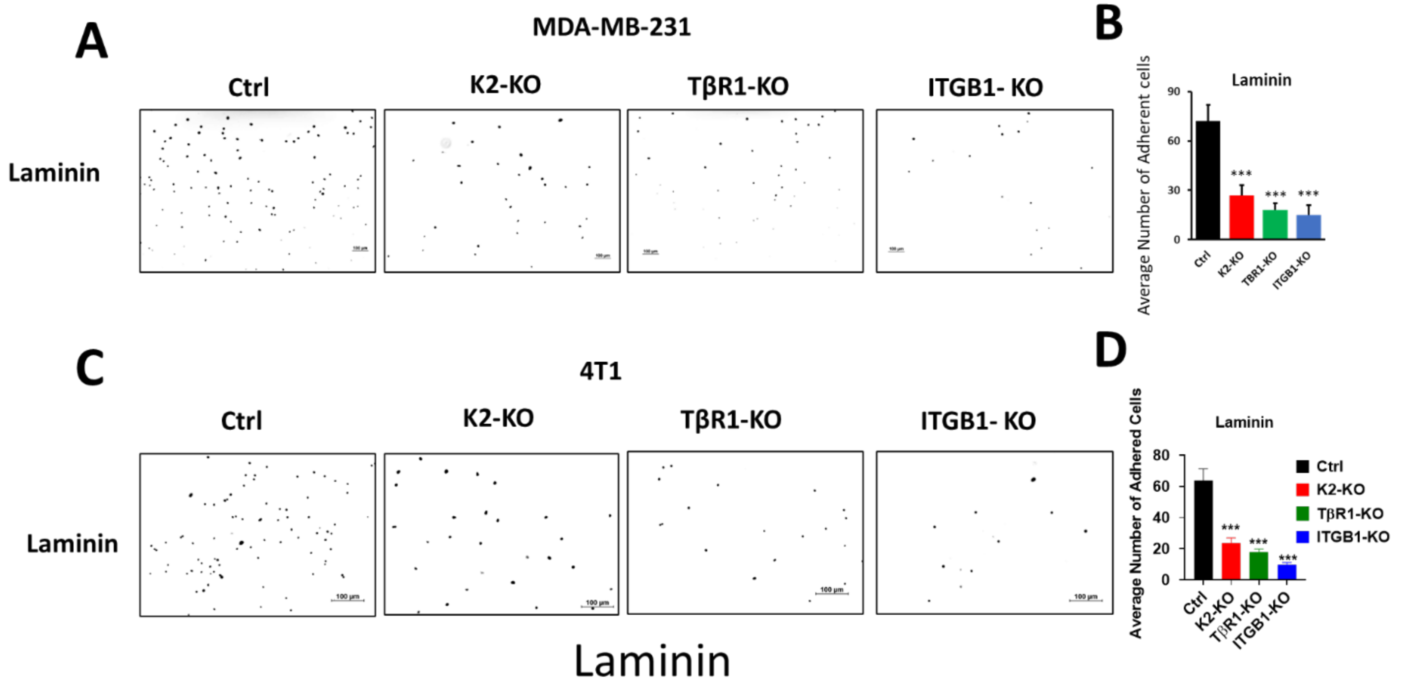

Figure. S6.

Representative confocal microscopy images of DAPI-stained MDA-MB-231 (A) 4T1 (C) cells and their K2-KO, TβR1-KO and ITGB1-KO derivatives that adhered to Laminin-coated coverslips. Scale bar :100 μm. (B&D)) Quantification of adherent cells. Data are the mean ± SD ($n = 3$, ***$p < 0.001$, Student's t test)

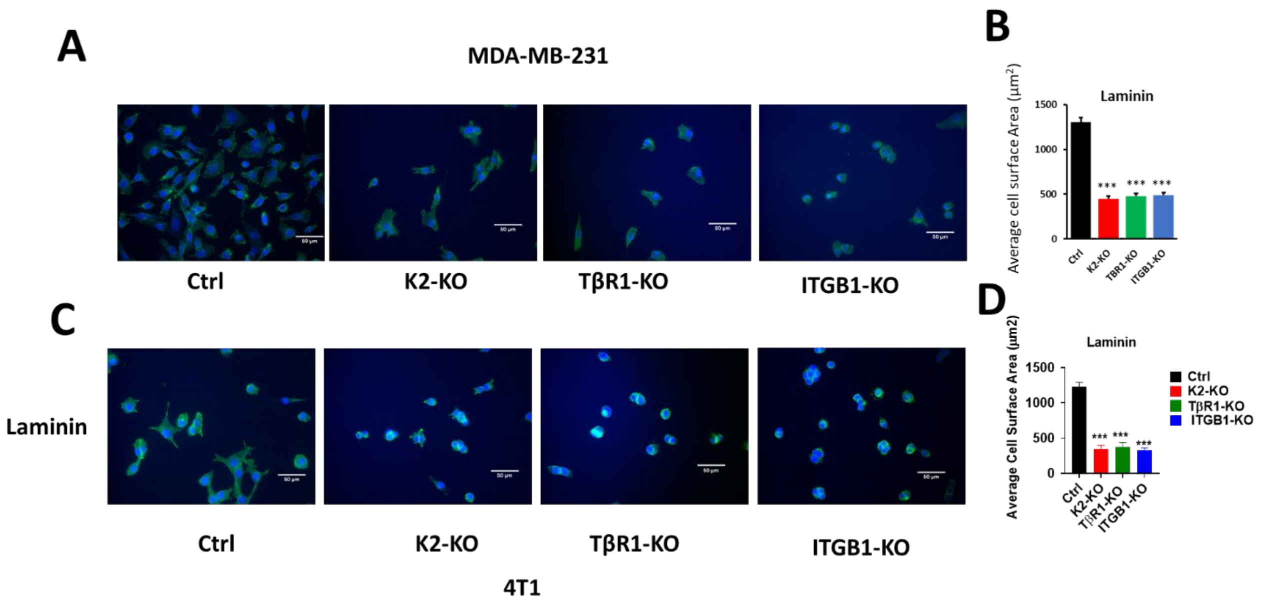

Figure. S7.

(A) Representative confocal microscopy images of MDA-MB-231 (A) 4T1 (C) cells and their K2-KO, TβR1-KO and ITGB1-KO derivatives that spread on Laminin-coated coverslips. Cells were stained phalloidin for actin (green). Nuclei were counterstained with DAPI (blue) Scale bar :50 μm. (B&D) Quantification of adherent cells (cell surface). Data are the mean ± SD (n = 3, ***p < 0.001, Student's t test).

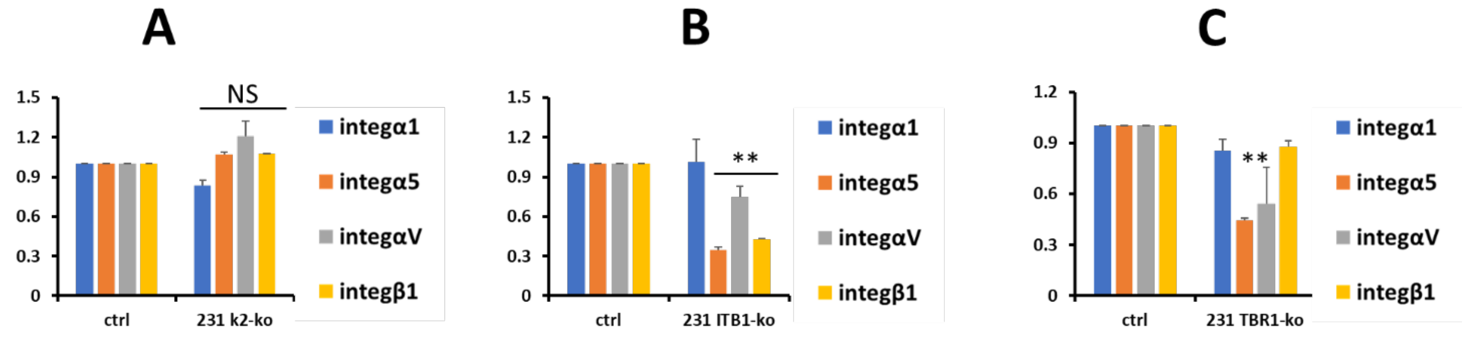

Figure. S8.

qt-RT-PCR quantification of mRNA levels the indicated Integrin-α subunits in control MDA-MB-231 cells and their K2-KO derivatives (A) ITGB1-Ko derivatives (B) and TBRI-KO derivatives (C). Data are the mean ± SD (n = 3, **p < 0.01, Student's t test).

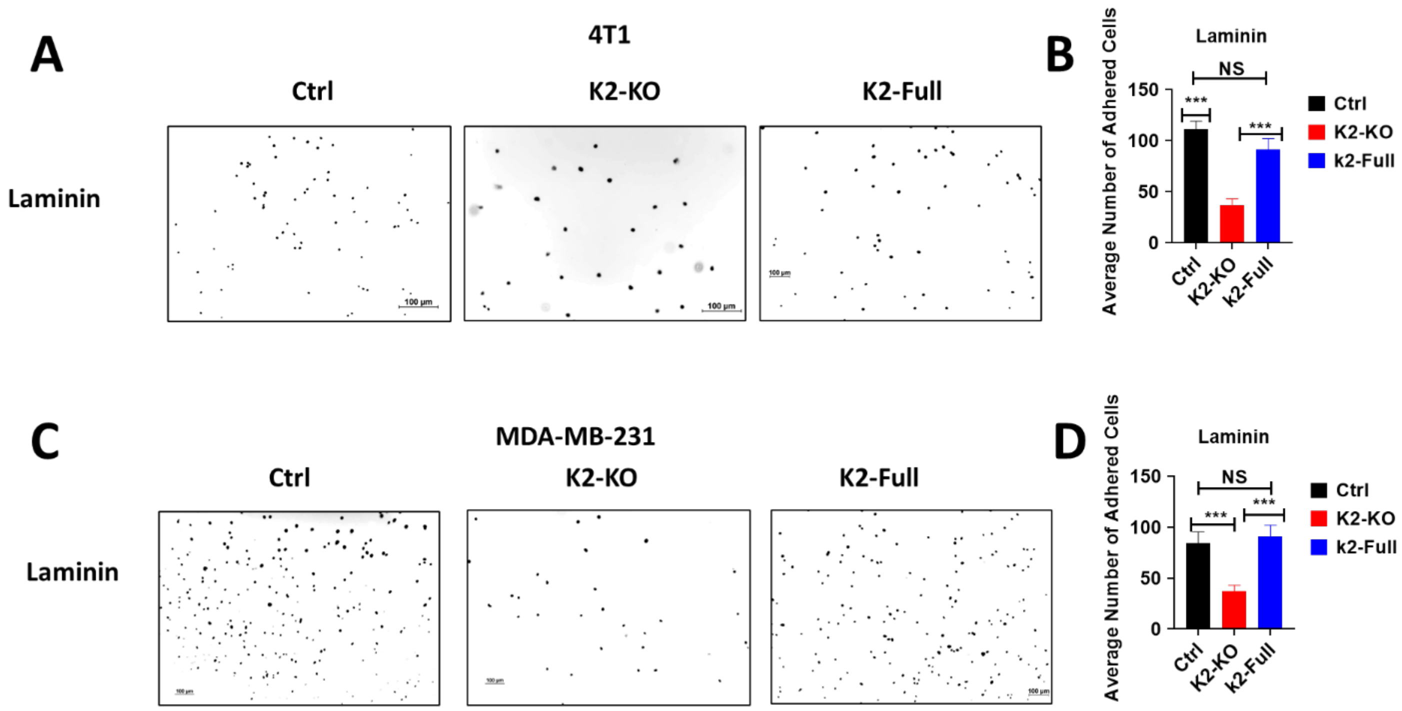

Figure. S9.

Representative confocal microscopy images of DAPI-stained MDA-MB-231 (A) and 4T1 cells (C) and their K2-KO and K2-Full derivatives that adhered to Laminin-coated coverslips. Scale bar :100 µm. (B and D) Quantification of adherent cells from each group. Data are the mean ± SD (n = 3, ***p < 0.001, Student's t test).

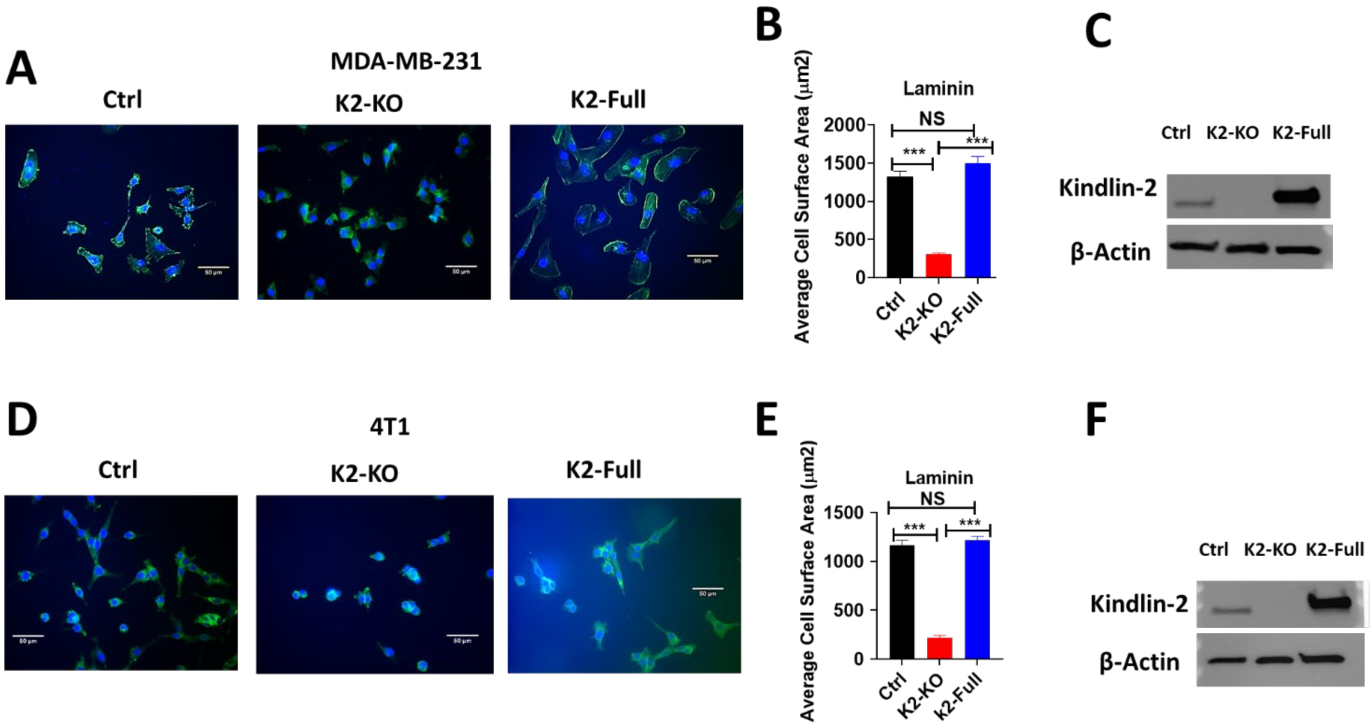

Figure. S10.

Representative confocal microscopy images of MDA-MB-231 (A) 4T1 cells (D) and their K2-KO and K2-Full derivatives that spread on Laminin-coated coverslips. Cells were stained with phalloidin for actin (green). Nuclei were counterstained with DAPI (blue). Scale bar :50 µm. (B and E) Quantification of adherent cells (cell surface). Data are the mean ± SD (n = 3, ***$p < 0.001$, Student's t test). (C and F) Representative WB of cell lysates from MDA-MB-231 (C) or 4T1 cells (F) and their K2-KO and K2-Full derivatives, that were probed with anti-Kindlin-2 antibody. β-Actin was used and loading control.

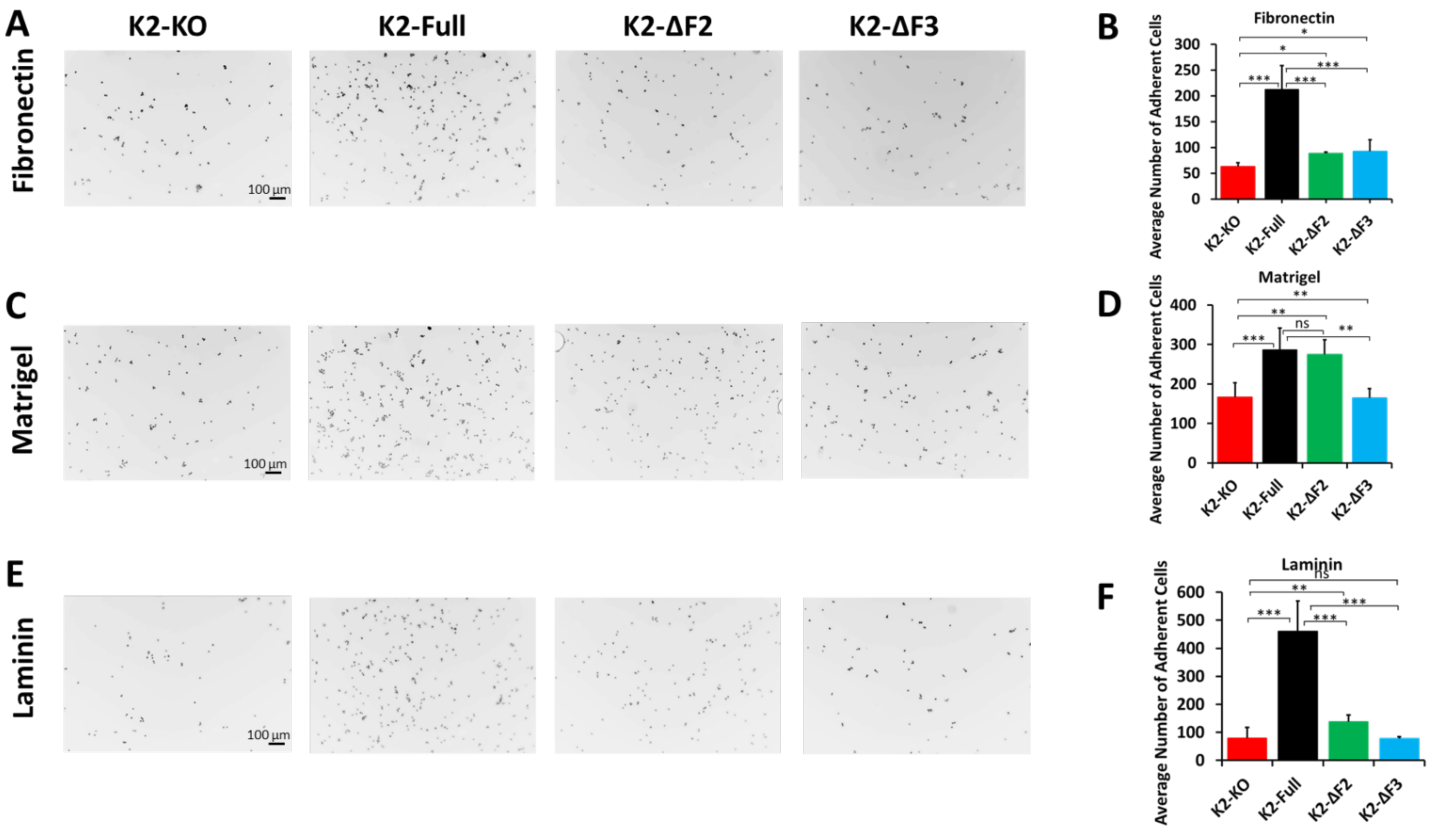

Figure. S11.

Representative confocal microscopy images of DAPI-stained K2-KO 4T1 cells and their K2-Full, K2-ΔF2 and K2-ΔF3 derivatives that adhered to Fibronectin- (A), Matrigel (B) or Laminin-coated coverslips. Scale bar :100 µm. (B, D and F) Quantification of adherent cells from each group. Data are the mean ± SD ($n = 3$, *$p < 0.05$, **$p < 0.01$, ***$p < 0.001$, Student's t test).

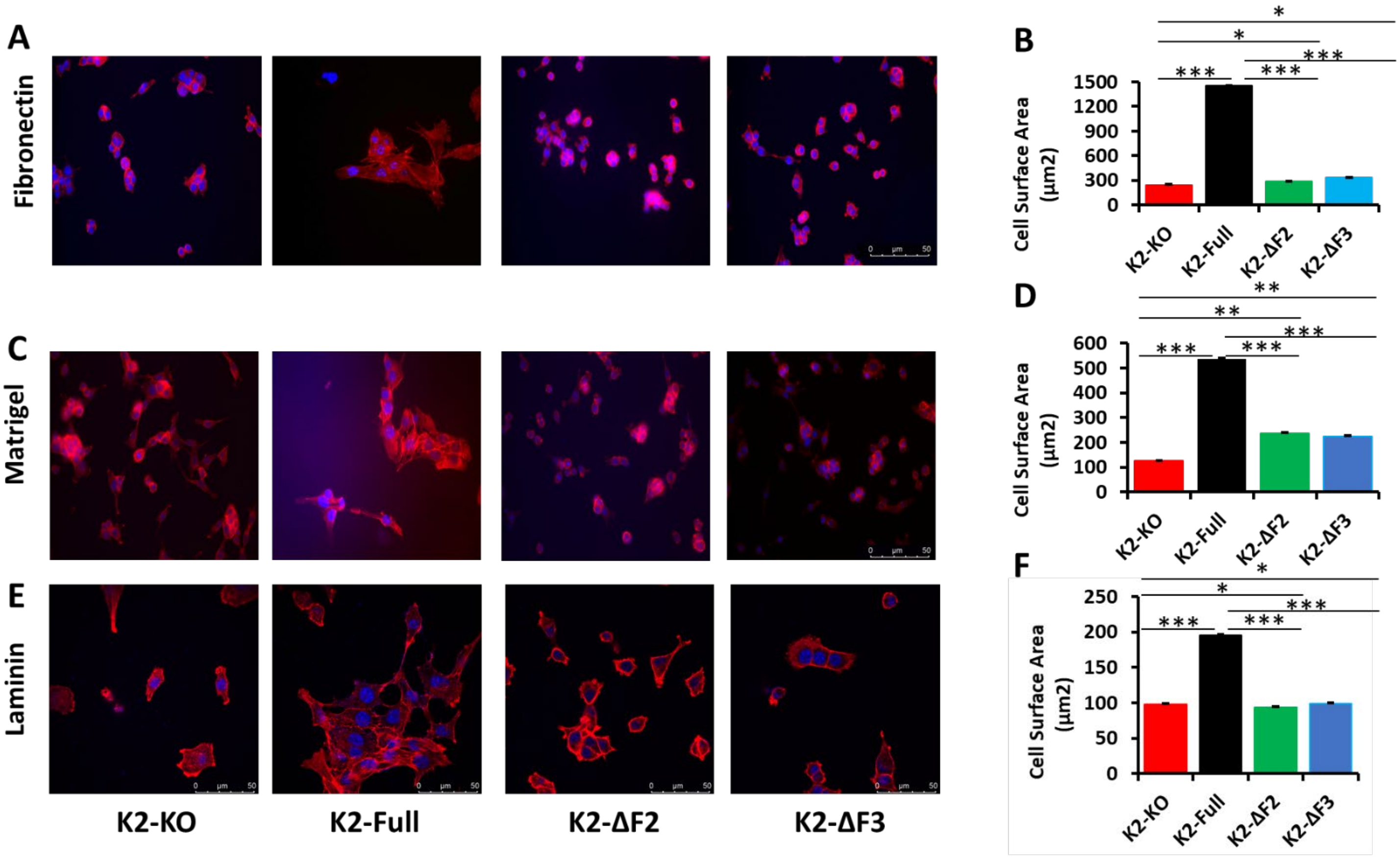

Figure. S12.

Representative confocal microscopy images of DAPI-stained K2-KO 4T1 cells and their K2-Full, K2-ΔF2 and K2-ΔF3 derivatives that adhered to Fibronectin- (A), Matrigel (B) or Laminin-coated coverslips. Cells were stained with phalloidin for actin (green). Nuclei were counterstained with DAPI (blue) Scale bar :100 µm. (B, D and F) Quantification of spread cells from each group. Data are the mean ± SD (n = 3, *p < 0.05, **p < 0.01, ***p < 0.001, Student's t test).

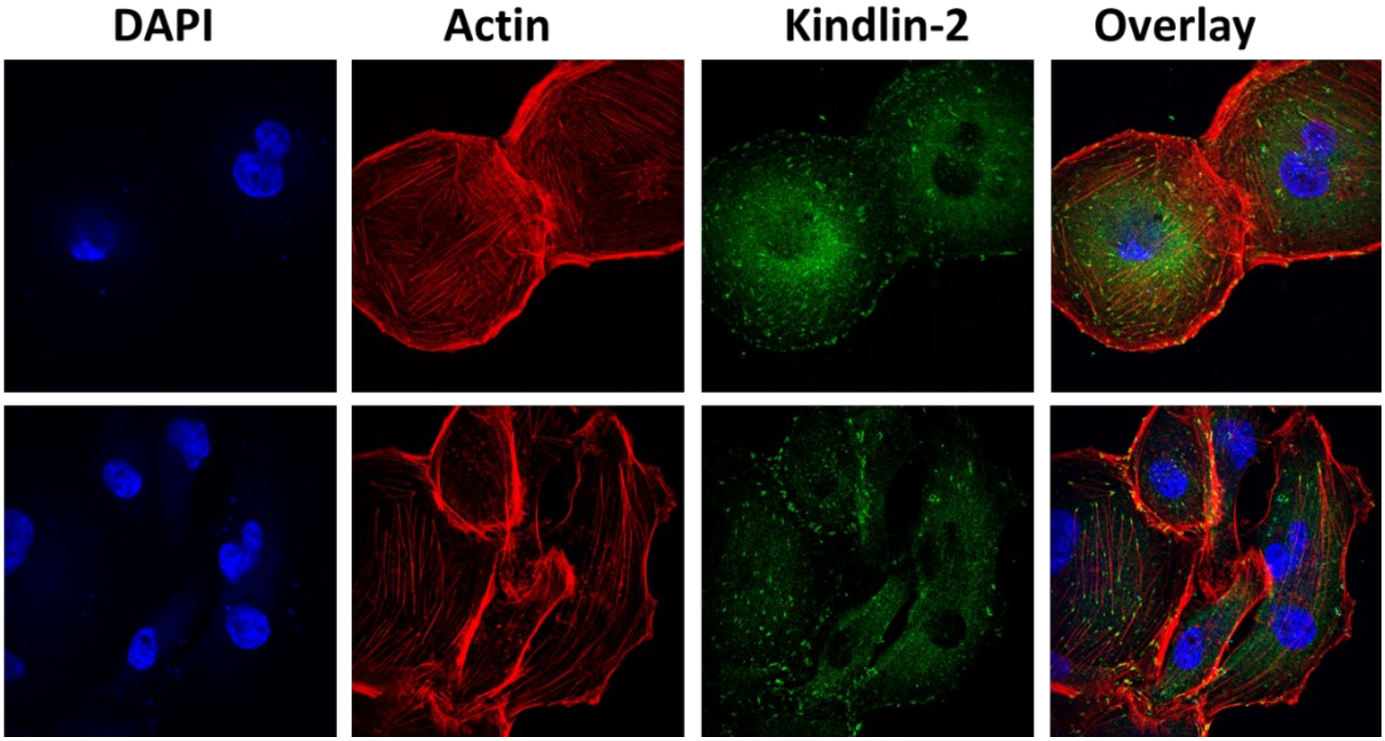

Figure. S13.

Representative confocal microscopy images of MDA-MB-231 cells that were stained with phalloidin for Actin (red) and antibodies against Kindlin-2 (green). Cell nuclei were counter stained with DAPI (blue). The staining shows the localization of Kindlin-2 in the focal adhesion complexes.

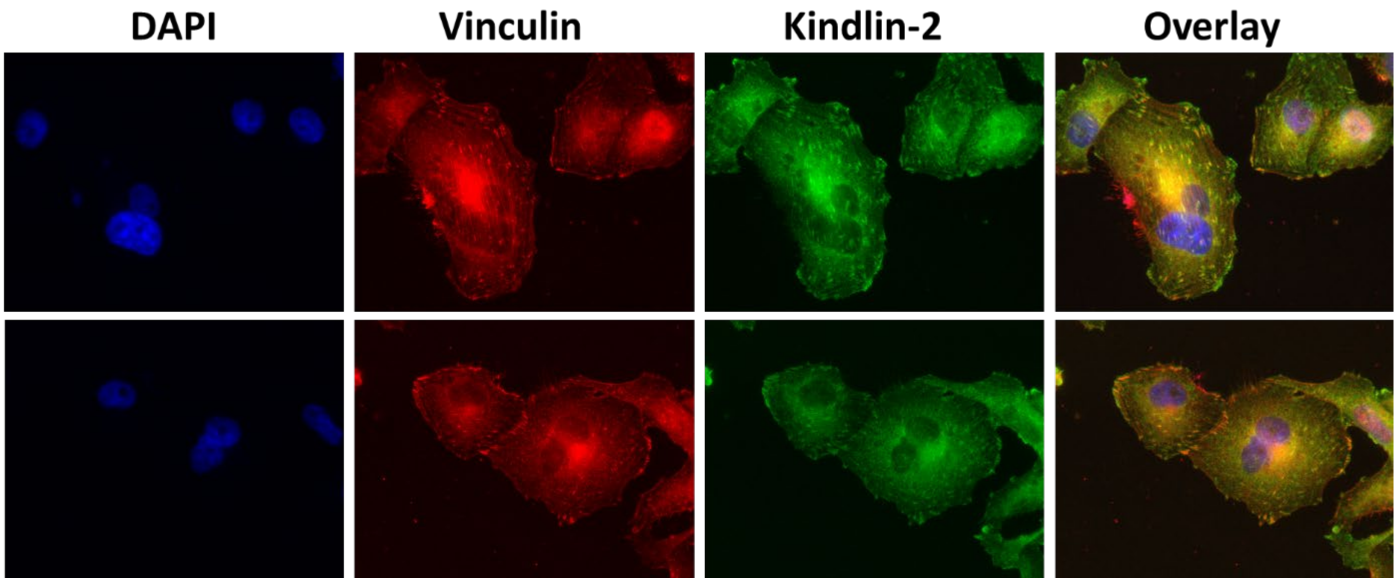

Figure. S14.

Representative confocal microscopy images of MDA-MB-231 cells that were stained with antibodies against Vinculin (red) and Kindlin-2 (green). Cell nuclei were counter stained with DAPI (blue). The staining shows the co-localization of Kindlin-2 and Vinculin in the focal adhesion complexes.

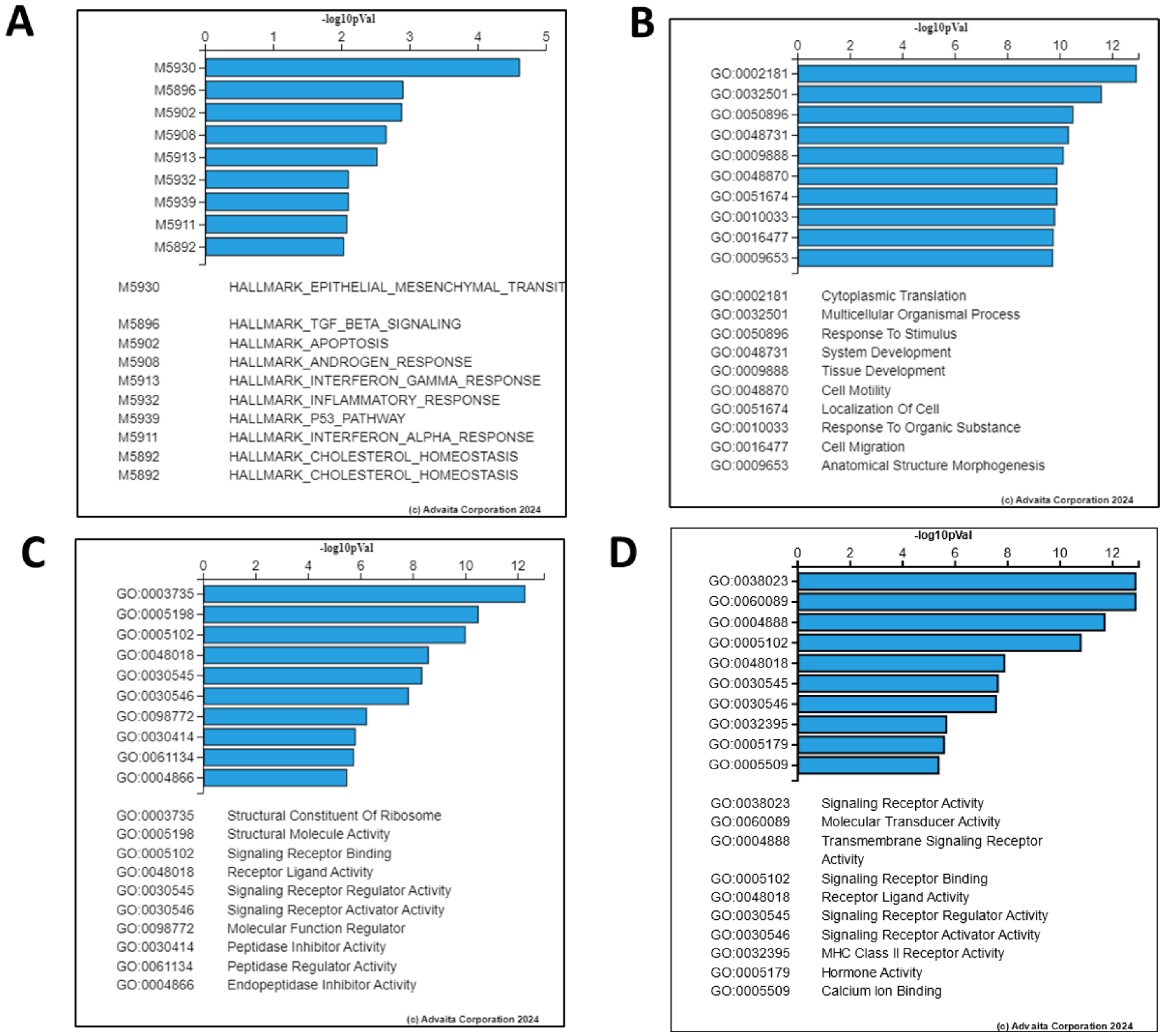

Figure. S15.
Pathway analyses using *iPathwayGuide* (ipathwayguide.advaitabio.com) of the RNA-seq data obtained from MDA-MB-231 cells and their K2-KO derivatives showed several significantly deferentially activated pathways as a result of loss of Kindlin-2 expression and demonstrating the potential involvement of Kindlin-2 in a range of oncogenic pathways. (A) Hallmark Signatures. (B) Gene Ontology (GO) Biological Processes. (C) GO Cellular Components. (D) GO Molecular Functions. The RNA-seq data was previously reported in Sossey-Alaoui et al., Cell Death Dis. 2019 Jul 15;10(8):539. PMID: 31308359.

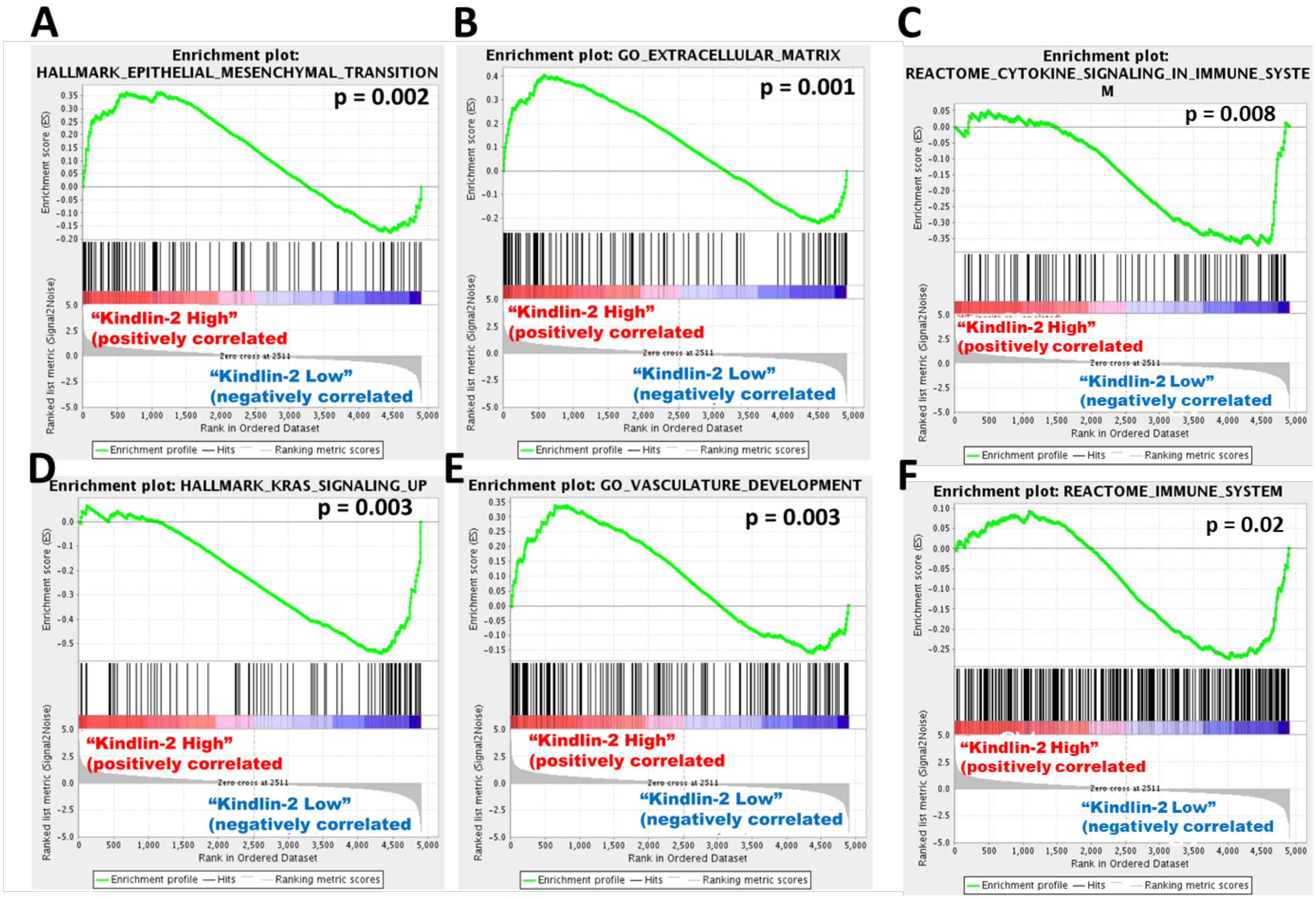

Figure. S16.
Gene Set Enrichment Analysis (GSEA) of the RNA-seq data obtained from MDA-MB-231 cells and their K2-KO derivatives showed that Kindlin-2 expression levels correlate with enrichment of gene sets that are involved in Epithelial-Mesenchymal Transition (A), Extracellular Matrix (B), Cytokine Signaling (C), KRAS signaling (D), Vasculature Development (E), and anti-tumor immune response (F). These data also demonstrate the potential involvement of Kindlin-2 in a range of oncogenic pathways. The RNA-seq data was previously reported in Sossey-Alaoui et al., Cell Death Dis. 2019 Jul 15;10(8):539. PMID: 31308359.

Name	Symbol	Sequence of sgRNA	Reference
Human-K2	H-sgRNA-1	$5^{/}$-AGGCGTGATGCTTAAGCTGG-$3^{/}$	SYNTHEGO
Human-K2	H-sgRNA-2	$5^{/}$-GGTATACTTGCTGTCAGTCA-$3^{/}$	//
Mouse-K2	M-sgRNA-1	$5^{/}$-CGGGGAGGTGCACATCGGAG-$3^{/}$	//
Mouse-K2	M-sgRNA-2	$5^{/}$-GTGACCGGGGAGGTGCACAT-$3^{/}$	//
Human-ITGβ1	H-sg RNA-1	$5^{/}$-UGUUCCUUUGCUACGGU-$3^{/}$	//
Human-ITGβ1	H-sg RNA-2	$5^{/}$-GACAUAGAAAAUCCCAG-$3^{/}$	//
Human-ITGβ1	H-sg RNA-3	$5^{/}$-UAGACAUUUUUACAGGA-$3^{/}$	//
Mouse- ITGβ1	M-sg RNA-1	$5^{/}$-CUUGCUAUGGAAACUC-$3^{/}$	//
Mouse-ITGβ1	M-sg RNA-2	$5^{/}$-GAGUAAUGUCUUCUGGC-$3^{/}$	//
Mouse-ITGβ1	M-sg RNA-3	$5^{/}$-AUAGUUGAGAGCCUCU-$3^{/}$	//
Human-TGFβR1	H- sg RNA-1	$5^{/}$-UCAGUAAAGUCAUCACC-$3^{/}$	//
Human-TGFβR1	H- sg RNA-2	$5^{/}$-CAUCUCACUCAUGUUGA-$3^{/}$	//
Human-TGFβR1	H- sg RNA-3	$5^{/}$-CUUCAUUUGGCACUCGA-$3^{/}$	//
Mouse-TGFβR1	M-sg RNA-1	$5^{/}$-GUGCCUCUGAAAUGAA-$3^{/}$	//
Mouse-TGFβR1	M-sg RNA-2	$5^{/}$-GAAUGACAGUGCGGUUA-$3^{/}$	//
Mouse-TGFβR1	M-sg RNA-3	$5^{/}$-CUGGCAGCUGUCAUUGC-$3^{/}$	//

Table S1. Sequence of sgRNAs used for CRISPR/Cas9-mediated knockout of the genes involved in this study.

Name & Symbol	Catalog No.	GeneGlobe ID	Gene Id	Reference position
Fermitin family member 2, FERMT2 [Human]	330001	PPH15416B-200	10979	1128
Fermitin family member 2, FERMT2 [Mouse]	3330001	PPM33282A	210018	2101
Glyceraldehye-3-phosphate dehydrogenase, GAPDH [Mouse]	330001	PPM02946E	309092	499
Integrin beta1, ITGβ1 [Human]	330001	PPH00650B	643813	2304
Integrin beta1, ITGβ1 [Mouse]	3330001	PPM03668D	263396	2744
Transforming growth factor beta receptor1, TGFβR1 [Human]	330001	PPH00237C	494622	1183
Transforming growth factor beta receptor1, TGFβR1[Mouse]	330001	PPMO3072C	197552	1570

Table S2. qt-RT-PCR primers information
